# Analyzing time-to-first-spike coding schemes: A theoretical approach

**DOI:** 10.3389/fnins.2022.971937

**Published:** 2022-09-26

**Authors:** Lina Bonilla, Jacques Gautrais, Simon Thorpe, Timothée Masquelier

**Affiliations:** ^1^CERCO UMR5549, CNRS – Université Toulouse III, Toulouse, France; ^2^Centre de Recherches sur la Cognition Animale (CRCA), Centre de Biologie Intégrative (CBI), Université de Toulouse, Toulouse, France; ^3^CNRS, UPS, Toulouse, France

**Keywords:** spiking neural networks, temporal coding, time-to-first-spike coding, rank-order coding, N-of-M coding

## Abstract

Spiking neural networks (SNNs) using time-to-first-spike (TTFS) codes, in which neurons fire at most once, are appealing for rapid and low power processing. In this theoretical paper, we focus on information coding and decoding in those networks, and introduce a new unifying mathematical framework that allows the comparison of various coding schemes. In an early proposal, called rank-order coding (ROC), neurons are maximally activated when inputs arrive in the order of their synaptic weights, thanks to a shunting inhibition mechanism that progressively desensitizes the neurons as spikes arrive. In another proposal, called NoM coding, only the first *N* spikes of *M* input neurons are propagated, and these “first spike patterns” can be readout by downstream neurons with homogeneous weights and no desensitization: as a result, the exact order between the first spikes does not matter. This paper also introduces a third option—“Ranked-NoM” (R-NoM), which combines features from both ROC and NoM coding schemes: only the first *N* input spikes are propagated, but their order is readout by downstream neurons thanks to inhomogeneous weights and linear desensitization. The unifying mathematical framework allows the three codes to be compared in terms of discriminability, which measures to what extent a neuron responds more strongly to its preferred input spike pattern than to random patterns. This discriminability turns out to be much higher for R-NoM than for the other codes, especially in the early phase of the responses. We also argue that R-NoM is much more hardware-friendly than the original ROC proposal, although NoM remains the easiest to implement in hardware because it only requires binary synapses.

## 1. Introduction

The last decade has seen an explosion in the use of neural networks for demanding AI problems that include computer vision, speech and audio processing, and natural language processing. Indeed, neural networks trained with Deep Learning are now state of the art in many domains. All such systems can be thought of as “neuromorphic” in that they involve large networks of neuron-like elements with connections that resemble the synapses of biological brains. However, there is currently an intense debate about whether future systems will need to include additional neuromorphic features. One key difference between these state-of-the-art AI systems and biology is how information is represented. Artificial systems typically perform calculations using floating-point variables to represent both the neuronal activation levels and the strength of synaptic connections. In contrast, real neurons send information as discrete all or none pulses—spikes. Is this difference important? Spiking Neural Networks (SNNs) are becoming increasingly popular, especially for low-power embedded systems. But many mainstream researchers consider that this difference is essentially irrelevant. Many assume that neurons send information using a firing rate code in which the neuron's activation level is represented by the number of spikes emitted in a given time window. If that was the case, replacing the firing rate with a floating-point number is a perfectly reasonable strategy. However, it has been argued that this sort of firing rate code would be intrinsically very inefficient because you would need a lot of spikes to encode information with any degree of accuracy (Gautrais and Thorpe, 1998). For example, suppose that we wanted to represent the activation level with a precision of 8-bits. To do this using a conventional rate code would mean waiting long enough for the neuron to emit 255 spikes when maximally activated—and this would mean waiting for a second or more to make even the most basic decisions. This very low efficiency has led some researchers to rule out spike-based coding schemes. They point out that it is much simpler, and much more accurate, to represent information as a floating-point number that can be transmitted in a single clock cycle *via* a 32-bit bus.

You could argue that there are alternative ways of implementing a firing rate based code that are much faster. For example, rather than sending an 8-bit activation level using a single neuron that emits between 0 and 255 spikes in a given time window, you could have 255 neurons in parallel, each of which only needs to emit at most one spike in, say, 10 ms. But this sort of population rate coding scheme would also be very inefficient because it would need very large numbers of neurons.

You might also argue that it is possible to estimate the instantaneous firing rate of a neuron by looking at the interval between two spikes. An interspike interval of exactly 4.0 ms would correspond to an instantaneous firing rate of 250 spikes/second. And, in such a case, the accuracy with which the underlying rate can be determined would be limited only by the temporal precision with which the neuron can emit spikes. If the precision was 0.1 ms, you could encode many different activation values in 25 ms. But while possible in principle, such a scheme would require very complex mechanisms to decode as well as being unusable until the neuron has emitted 2 spikes.

It would appear that the fundamental problem here is that researchers have apparently been assuming that spike-based coding has to be some sort of rate coding scheme. But this is certainly not the case. Even the simplest neuronal models have the property that the time taken for a neuron to reach threshold depends on the intensity of the input. And this means that the latency of the first spike in response to a stimulus can be used as a code. Remarkably, variations in spike latency with input intensity were demonstrated in the very first recordings of activity in the optic nerve by Lord Edgar Adrian in Cambridge in the 1920s (Adrian, 1928). But this basic physiological fact was essentially ignored for several decades, before being demonstrated again by neurophysiological studies (Gollisch and Meister, 2008).

Once one accepts the idea that the timing of the first spike provides an alternative way to encode information—a scheme known as time-to-first spike coding (TTFS)—, there are a number of very interesting options that can be considered. In principle, you could use the latency at which a single neuron fires in response to an input to derive information about the intensity of the activation. For example, a neurophysiologist could use an oscilloscope to determine a neuron's latency. But this requires knowing precisely when the stimulus came on. Inside the brain, there is no way to know this. Hence, in this paper we consider an alternative strategy: looking across a population of neurons and determining the order in which they fire. Note that TTFS is not well-suited for dynamic inputs, since coding changes in the input requires additional spikes. We thus focus on static inputs, e.g., flashed images. For simplicity and hardware-friendliness, we also restrict ourselves to non-leaky neurons. A leak is useful to process dynamic inputs because the oldest inputs should be forgotten. Yet it is not required with the static inputs used in this paper.

Historically, TTFS was first proposed to explain the phenomenal speed of processing in the brain for certain tasks, such as object recognition (Thorpe and Imbert, 1989). More recently, TTFS has attracted much attention from the AI community (Mostafa, 2017; Rueckauer and Liu, 2018; Zhou et al., 2019; Kheradpisheh and Masquelier, 2020; Park et al., 2020; Sakemi et al., 2020; Zhang et al., 2020; Comsa et al., 2021; Mirsadeghi et al., 2021), because it can be efficiently implemented on low power event-driven neuromorphic chips (Abderrahmane et al., 2020; Nair et al., 2020; Srivatsa et al., 2020; Göltz et al., 2021; Liang et al., 2021; Oh et al., 2022), leveraging two key features. The first one is sparsity (Frenkel, 2021). Neurons fire at most once, but usually most neurons do not fire at all. Processing thus consumes very few spikes, and thus very little energy, because usually idle neurons do not consume much (Davies et al., 2018). The second one is time. If using event-driven processing, for example, address event representation (AER), time represents itself (Mead, 1990). Thus one can compute with time without ever storing timestamps. For example, a decision can be made based on the first neuron to fire in the readout layer. And this is possible even if the firing time difference is infinitesimally small. Conversely, a readout based on the activation levels requires storing these activation levels with high precision to be able to always distinguish the most active neuron.

It is worth mentioning that neurons are intrinsically sensitive to the timing of their inputs: shifting the input spike times obviously shifts the response time. But here, we consider additional mechanisms that allow neurons to respond selectively to certain input spike time patterns. For example, Rueckauer and Liu (2018), Sakemi et al. (2020), Srivatsa et al. (2020), and Zhang et al. (2020) used linearly increasing excitatory postsynaptic potentials, such that early spikes contribute more. To obtain a similar effect, Park et al. (2020) used a decaying dendritic kernel. Yet in this paper, we focus on spike-based, rather than time-based mechanisms: the input spikes' contribution depends on their arrival ranks rather than on their precise times. The idea is always that the first input spikes contribute more, while later input spikes contribute less, or not at all. This is implemented with a modulation function that decreases with the rank, for example, linearly or geometrically. The net contribution of each input spike to the neuron's potential is then the product of the modulation function with the synaptic weight. The modulation function can also have a cut-off so that the last spikes make no contribution at all.

Our main goal, below, is to lay the foundation of a mathematical framework in order to assess, from a theoretical point of view, the potential of such order-based TTFS coding schemes. As an illustration of this framework, the analysis will be performed upon three instances of such coding schemes: two previous proposals (Rank Order Coding and NoM coding) and a combination of both (Ranked-NoM Coding).

**Figure 1:**
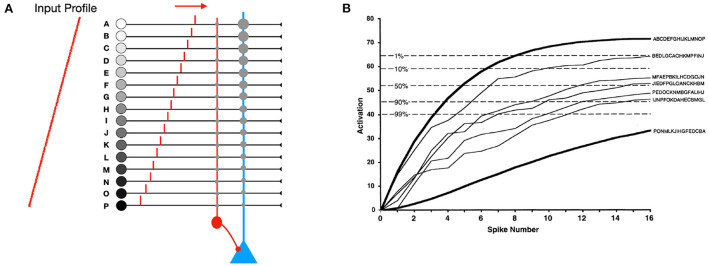
**(A)** Rank order coding (ROC) with *M* = 16 afferents. All the afferents fire exactly one spike. Here we show a neuron selective to the input spike order: A, B, ... , P. Its synaptic weights are linearly decreasing: *M* for input A, *M* − 1 for input B, and so on, down to 1 for input P. The modulation decreases geometrically with the input spike rank. In practice, this modulation could be implemented with shunting inhibition, as shown with the red inhibitory neuron. **(B)** The increase in activation level depends on the order of firing. Maximal activation occurs when the inputs fire in the order of the weights (A, B, ... , P). Activation is minimal when the order is reversed. Intermediate lines correspond to 5 randomly selected input patterns chosen from the 16! = 20, 922, 789, 888, 000 possible input spike orders. The five dotted lines specify the proportion of such random patterns that will exceed a given final activation level. Modified from Thorpe and Gautrais (1998).

Rank Order Coding (ROC) was an early proposal (Thorpe and Gautrais, 1998). With ROC, all the *M* afferents of a neuron fire a spike (Figure 1). The modulation is a real number which decreases geometrically with the input spike rank. That means in particular that it is always strictly positive. The synaptic weights are *M*, *M*−1, ... 1. The final potential is maximal when input spikes arrive in the order of the weights: the first spike should arrive through the synapse with weight *M*, the second one through the synapse with weight *M* − 1, and so on.

**Figure 2:**
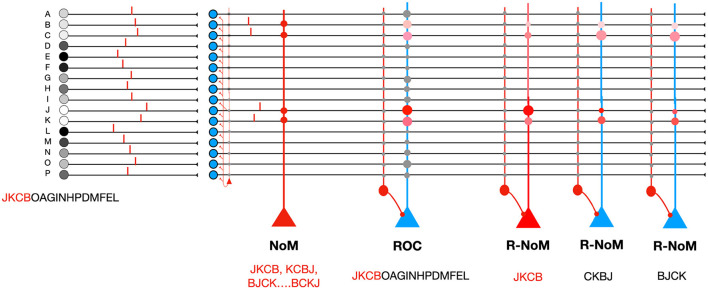
Comparison of different codes. On the left, the *M* = 16 afferents fire in the order JKCBOAGINHPDMFEL, but a 4-winner-take-all mechanism only lets the *N* = 4 first spikes through. NoM coding: the readout neuron uses binary weights: *W* = 4 ones, and *M* − *W* = 12 zeros. The final potential reaches the maximal value of 4 if the *N* first spikes correspond to the *W* non-zero weights. The order of these 4 first spikes does not matter. Rank Order coding **ROC**.: the neuron is set up to respond maximally to the order JKCBOAGINHPDMFEL, even though here only the 4 input spikes are propagated. Ranked-NoM coding **R-NoM**:: we show three readout neurons that are selective to three different orders for the 4 first spikes, among the 4! = 24 possible orders, thanks to graded weights and modulations, both in {1, 2, 3, 4}.

N-of-M (NoM) coding is another proposal, in which only the *N* first spikes among *M* afferents are propagated (Furber et al., 2004; Thorpe et al., 2019). This first spike pattern can be read out by neurons with binary weights (Figure 2): *W* = 4 ones, and *M* − *W* = 12 zeros. With random inputs, the final potential has a hypergeometric distribution with *N* draws from a population of size *M* containing *W* successes—or, equivalently, *W* draws from a population of size *M* containing *N* successes (Furber et al., 2004).

For this paper, we have also designed a third type of coding scheme, that we call “Ranked-NoM” (R-NoM) coding, and which incorporates features of both ROC and NoM coding (Figure 2): only the *N* first spikes among *M* afferents are propagated, but readout neurons can be selective to a particular order of the *N* spikes thanks to inhomogeneous weights, and a decreasing modulation function. Later on, we came across an article by Furber et al. (2007) where a similar proposal has been explored in the context of sparse distributed memory (SDM) research. Below, both the weights and the modulation decrease linearly, although other schemes could also be explored using a similar approach (e.g., geometric series as in Furber et al., 2007).

All these codes have been formalized in our unifying mathematical framework that involves:

- A set of weights, which can be homogeneous (as in NoM), or decreasing, either linearly (as in original ROC), or geometrically. This set contains *W* non-zero weights.
- A modulation function which can be constant (as in NoM), or decreasing, either linearly, or geometrically (as in original ROC). This modulation can also have a cut-off, i.e., becomes zero after the first *N* spikes.

Our unifying framework allows comparing these codes in terms of discriminative power. We introduce a discriminability measure that quantifies how much more a neuron responds to its preferred pattern than to random inputs. The unifying mathematical framework also allows tuning the parameters of the codes in order to optimize their discriminative power.

We conclude that Ranked-NoM Coding with linearly decreasing modulation and weights offer a particularly interesting compromise between discriminative power and hardware-friendliness.

The paper is organized as follows: the Section 2 briefly introduces the unifying mathematical framework and the discriminability measure. Then, it gives the main analytical formulas for the discriminability of R-NoM, NoM, and ROC, but not their derivations, which can be found in the Supplementary material. Next, we report a numerical study in which we explored the speed-accuracy trade-off for the three different codes. Finally, a brief Discussion summarizes the main results and gives some perspectives.

## 2. Results

### 2.1. Mathematical translation of the three coding schemes

The goal is to measure the discriminability power of these codes. We define a measure of selectivity (Equation 2.7) which quantifies how much more the neuron responds to its preferred pattern than to random stimuli.

We first define a random experiment for the spikes generated by *M* neurons (see Supplementary Section 1.2). For a given stationary stimulus, each of the *M* input neurones emits one spike. Input patterns will then translate into vectors of size *M*. We denote Λ the ascending lexically ordered set of the possible permutations over the set M={0,…,M-1}. Cardinality of Λ is then *M*!. We define an application R that takes values in $D_{K}=\left\{1,2,..,M!\right\}$ (ranks of input order in Λ) and returns a vector ${\text{r}}^{k}=R\left(k\right)$ in Λ.

To randomly generate sets of input patterns, we define a discrete random variable *K* over $D_{K}$. We can then consider X=R(K) as a random vector, and all possible outputs are collected in $D_{\text{X}}=\text{Λ}$. We consider that all input orders have the same probability to occur.

By construction, each component *X*_*i*_ is a discrete random variable taking values from the set $D_{X_{i}}=\left\{0,1,\ldots ,M-1\right\}$ with marginal probability distribution ${\text{P}}_{X_{i}}\left(r\right)=\frac{1}{M},$ and multivariate joint probability distribution ${\text{P}}_{X_{1}X_{2}\ldots X_{M}}=\frac{1}{M!}.$ *X*_*i*_ are identically distributed and they are not independent since realizations of **X** are permutations from a unique set of values, the one prescribed by the coding scheme, which implies correlation, so that: Cov(*W*_*i*_, *W*_*j*_) ≠ 0.

This input order is transformed into a vector of weights. For this, we transform the random variable **X** in a deterministic way by defining the affine transformation **W** = Φ(**X**):


(2.1) $$\begin{array}{l} \text{Φ}\left(\text{X}\right)=M-\text{X}=\text{W} \end{array}$$

The marginal and joint probability distributions of the new random variable *W*_*i*_ are determined from the probability distributions of *X*_*i*_ by the change of variables theorem in multivariate calculus. At this stage, the random experiment is fully defined by the random variable **X**, taking values in Λ, and the bijective function Φ.

We denote Ω the set of the weights vectors. Ω is the base to establish the support of each coding scheme. For this, we define, for each scheme *C*, a vector-value function Φ_*C*_ from Ω to Ω_*C*_ and we use the term *score vector* to denote elements in Ω_*C*_.

For ROC (denoted by *R*), the function Φ_*R*_ is the identity function and so its cardinality is *M*!.

For Ranked-NoM Coding (denoted by *H*), we build the scores-support Ω_*H*_ using a function which depends on the parameter W:


(2.2) $$\begin{array}{l} {\text{Φ}}_{H}\left(\text{w}\right)=\max\left(0,\text{w}-M+W\right) \end{array}$$

Note that Φ_*H*_ maps different permutations onto the same vector permutation. Hence, a subset of vectors that are pure internal permutations among negative or null values will map to the same element of Ω_*H*_. Since the cardinality of these subsets is the number of permutations of the M-W null elements, the cardinality of Ω_*H*_ is:


(2.3) $$\begin{array}{l} |{\text{Ω}}_{H}|=\frac{M!}{\left(M-W\right)!} \end{array}$$

For NoM coding (denoted *F*), we define the scores-support Ω_*F*_ from the scores-support Ω_*H*_ by the compositions of the indicator function **1**_*A*_ with Φ_*H*_. Thus we have


(2.4) $$\begin{array}{l} {\text{Φ}}_{F}(w)=1_{A}({\text{Φ}}_{H}(w))=1_{A}(\max(0,w-M+W)) \end{array}$$

By the indicator function, the vectors in Ω_*H*_ get converted into vectors of ones and zeros. As a consequence, the support Ω_*F*_ of NoM is reduced because the order is no longer important. Then, we divide by the number of ways you can arrange W numbers, which is W!. Thus, the cardinality of Ω_*F*_ is:


(2.5) $$\begin{array}{l} |{\text{Ω}}_{F}|=\frac{\left|{\text{Ω}}_{H}\right|}{W!}=\frac{M!}{W!(M-W)!}=\left(\begin{array}{c} M\\ W \end{array}\right) \end{array}$$

Having defined the scores vectors for each coding scheme by their scores-support; Ω_*H*_, Ω_*F*_ and Ω_*R*_, we can establish the probability and statistics to get the first two moments of the weights for each coding scheme (see Supplementary material).

Next, we define, for each scheme, a *modulations vector* ${\text{v}}_{C}^{1}={\text{Ψ}}_{C}\left[\text{Φ}\left(R\left(1\right)\right)\right]$, considering that, for the neuron under consideration, the preferred pattern corresponds to the first input pattern in Λ. For ROC, it depends on a modulation parameter *m* ∈ {1/*n*:*n* ∈ ℤ, *n* ≠ 1}, with ${\text{v}}_{R}^{1}=\left(m^{0},m^{1},m^{2},...,m^{M}\right)$. For Ranked-NoM, Ψ_*H*_ ≡ Φ_*H*_ (2.2), and for the NoM scheme Ψ_*F*_ ≡Φ _*F*_ (2.4).

Finally, we define an integration function — effectively equivalent to the membrane potential — which indicates how well the random scores vector matches the fixed modulations vector.

To formally translate intermediate states (i.e., before the propagation is over), we first define the gate function $G_{I}:\Xi_{C}\to {\mathbb{R}}^{M}$ which nullifies all components of the modulation vector for ranks beyond *I*. Then, over the first *I* inputs, the integration function *S*_*C*_(**w**,*I*) reads:


(2.6) $$\begin{array}{l} S_{C}(w,I)=\langle G_{I}\left(v_{C}^{1}\right),{\text{Φ}}_{C}(w)\rangle \end{array}$$

Given that Ranked-NoM and NoM are defined for values N<M, the final potential is obtained when I=N and we would have intermediate states only for values I<N. For ROC, the final potential is obtained when *I* = *M* and we would have intermediate states for all values *I* < *M*.

### 2.2. Coding schemes comparison

#### 2.2.1. Comparing discriminability

Since **w** is a random vector, then *S*_*C*_(**w**,*I*) is a random function. Let *S*_*C,I*_ denote the corresponding output random variable. Its distribution depends on the coding scheme. We compare the three coding schemes in terms of discriminative power, characterizing its distribution by the difference between its best possible value and its expected values, scaled by its variance.

**Definition 2.1**. We define discriminability *D*_*C*_(*I*) as:


(2.7) $$\begin{array}{l} D_{C}\left(I\right)=\frac{\max\left(S_{C,I}\right)-\text{E}\left[S_{C,I}\right]}{\sqrt{\text{Var}\left[S_{C,I}\right]}} \end{array}$$

where *I* ∈ ℤ and takes values for ROC in the interval [1, M] and for Ranked-NoM and NoM coding in the interval [1, N]. This discriminability is also known as the signal-to-noise ratio in other papers (Masquelier, 2018; Masquelier and Kheradpisheh, 2018; Jordan et al., 2021). Given that for values N<I<M, Ranked-NoM and NoM are not defined, we set those values to the final integration corresponding to each scheme.

The max(*S*_*C,I*_) (see Supplementary Sections 2.6.1, 3.6.1, and 4.6.1), for W>N, are given in Table 1.

**Table 1:** Formulas for the maximum value of integration *S*_*C,I*_ for each scheme.

	**max(*S*_*C,I*_)**
Ranked-NoM(H)	$WN\left(\frac{N+1}{2}\right)+\frac{N\left(1-N^{2}\right)}{6}$
NofM(F)	N
ROC(R)	$\frac{\left(1-m\right)\left(1+M\right)-\left(1-m^{M+1}\right)}{{\left(1-m\right)}^{2}}$

The expectation E[*S*_*C,I*_] and variance Var[*S*_*C,I*_] of integration at intermediate states of each scheme *C* depend on the mean μ_*W*_*C*__, variance Var*W*_*C*_ and covariance Cov_*C*_(*W*_*i*_, *W*_*j*_) of the scores for the corresponding coding scheme *C* (see Supplementary Sections for Ranked-NoM 2.7.1, 2.7.7, for NoM 3.7.1, 3.7.2, and for ROC 4.7.1, 4.7.2). Their full expressions are given in Table 2.

**Table 2:** Formulas for the expectation, variance and covariance of the scores random variable *W* for each scheme.

***C***	**μ_*W*_*C*__**	**Var*W*_*C*_**	**Cov_*C*_(*W*_*i*_, *W*_*j*_)**
Ranked-NoM(H)	$\frac{W\left(W+1\right)}{2M}$	$\mu_{W_{H}}\left(\frac{2W+1}{3}-\mu_{W_{H}}\right)$	$\frac{\mu_{W_{H}}}{M-1}\left(\mu_{W_{H}}-\frac{2W+1}{3}\right)$
NofM(F)	$\frac{W}{M}$	μ_*W*_*F*__(1−μ_*W*_*F*__)	$\mu_{W_{F}}\left(\frac{W-1}{M-1}-\mu_{W_{F}}\right)$
ROC(R)	$\frac{M+1}{2}$	$\mu_{W_{R}}\;\left(\frac{M-1}{6}\right)$	$\frac{\mu_{W_{R}}}{M-1}\;\left(\mu_{W_{R}}-\frac{2M+1}{3}\right)$

As a general pattern, we have the following non-linear functions,


(2.8) $$\begin{array}{l} \text{E}\left[S_{C,I}\right]=\lambda_{C}\;\mu_{W_{C}} \end{array}$$

(2.9) $$\begin{array}{l} \text{Var}\left[S_{C,I}\right]=\alpha_{C}\;\text{Var}W_{C}+\beta_{C}\;{\text{Cov}}_{C}\left(W_{i},W_{j}\right) \end{array}$$

where the constants λ_*C*_, α_*C*_ and β_*C*_ for each scheme are provided in Table 3.

**Table 3:** Formulas for the expectation and variance coefficients of the different Integration schemes.

***C***	**λ_*C*_**	**α_*C*_**	**β_*C*_**
Ranked-NoM(H)	$\frac{I\left(2N-I+1\right)}{2}$	N I(N-I+1)+	N I(I-1)(N-I+1)+
		$\frac{I\left(I-1\right)\left(2I-1\right)}{6}$	$\frac{I^{2}{\left(I-1\right)}^{2}}{4}-\frac{I\left(I-1\right)\left(2I-1\right)}{6}$
NofM(F)	*I*	*I*	*I* (*I*−1)
ROC(R)	$\frac{1-m^{I}}{1-m}$	$\frac{1-m^{2I}}{1-m^{2}}$	${\left(\frac{1-m^{I}}{1-m}\right)}^{2}-\frac{1-m^{2I}}{1-m^{2}}$

#### 2.2.2. Behavior of discriminability for final potential

Having established the complete expression of discriminability for the three schemes, we can now compare how they perform.

**Figure 3:**
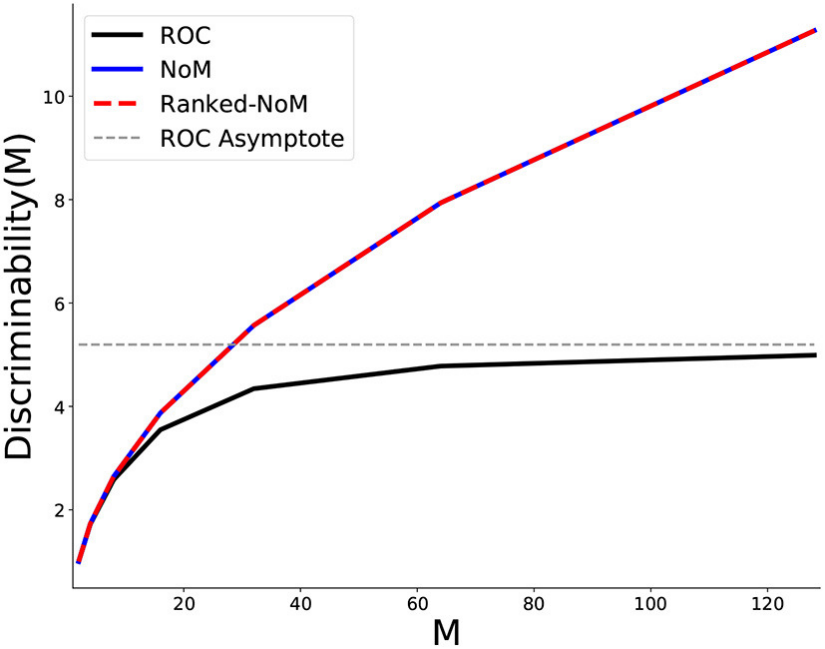
Behavior of the maximal discriminability as a function of the total number of inputs *M*. NoM and Ranked-NoM (set to W=N=M/2) converge to the same maximal values and so the two curves overlap. For these two schemes, the maximal discriminability scales as $D_{H}\left(M\right)=D_{F}\left(M\right)=\sqrt{M-1}$ (proof to be found in Supplementary material). Hence, discriminability is not limited, and adding inputs will always improve it. By contrast, maximal discriminability for ROC saturates at an asymptotic value (*Y*≃5.2 for *m* = 0.8 here).

We first illustrate how the total number of available inputs (*M*) affects discriminability (Figure 3).

Setting N=W=M/2 for Ranked-NoM and NoM codes, we get the same function for both schemes (see Supplementary Sections 2.8 and 3.8):


(2.10) $$\begin{array}{l} D_{H}\left(M\right)=\sqrt{M-1} \end{array}$$

For ROC, we found (see Supplementary Section 4.8):


(2.11) $$\begin{array}{l} \underset{M\to \infty }{\lim}D_{F}\left(M\right)=\frac{\sqrt{3}}{1-m}\sqrt{1-m^{2}} \end{array}$$

For *m* = 0.8, the function *Y* = *D*_*F*_(*M*) has a horizontal asymptote in *Y*≃5.2:


(2.12) $$\begin{array}{l} \underset{M\to \infty }{\lim}D_{F}\left(M\right)=\frac{\sqrt{3}}{1-0.8}\sqrt{1-0.8^{2}}≃5.2 \end{array}$$

In light of these behaviors, we propose that Ranked-NoM and NoM are to be preferred over ROC.

#### 2.2.3. Behavior of discriminability during propagation

**Figure 4:**
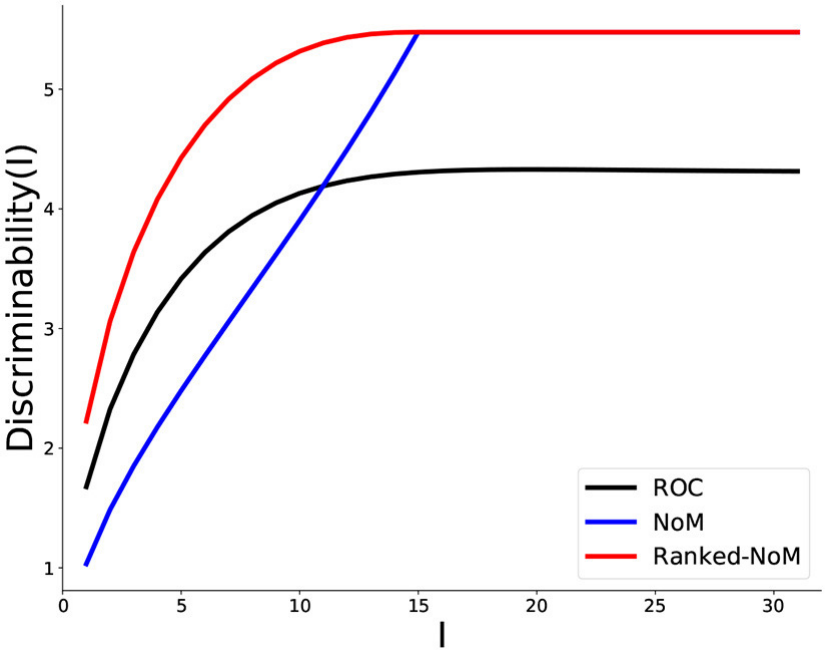
Comparison of discriminability for the three coding schemes during propagation. The Discriminability, *D*_*C*_(*I*) (Equation 2.7) is reported as the number of inputs *I* builds up, for each coding scheme: ROC (black), NoM (blue), and Ranked-NoM (red). For ROC coding, the inputs *I* accumulate up to the maximal number (here, *M* = 31) while, in the two others, propagation stops beyond N=15 (in this case, we retain the value $D_{C}\left(N\right)$ for later values).

We now contrast, for a given *M* = 31, how discriminability increases as more and more inputs become available (namely, potential integration, Figure 4).

As shown above, discriminability saturates to the same value for Ranked-NoM and NoM (here, N=W), while, for ROC, it saturates at a lower value, which depends on the ROC-parameter *m* (here *m* = 0.8).

We also observe that NoM performs poorly early on since discriminability increases nearly linearly, while both ROC and Ranked-NoM increase more like an exponential relaxation to the final value.

In contrast to NoM, Ranked-NoM Coding then displays a much faster increase in discriminability in the early phase of input integration and reaches a higher value than ROC.

In this regard, Ranked-NoM displays the best performance, with a high discriminability for the very early inputs.

#### 2.2.4. Exploring the speed-accuracy trade-off through simulations

Importantly, our discriminability measure (Equation 2.7) is based on the unconstrained membrane potential, i.e., ignoring the threshold. But of course, in a real scenario, a threshold is needed, especially for neurons in the hidden layers (otherwise, they will not fire!). When choosing a threshold, a high value:

- Ensures that the probability of reaching it with random input (which may be seen as a false alarm, FA) is low.
- Causes a longer latency even when the preferred pattern is given as input.

Conversely, a low threshold does the opposite (shorter latency but higher FA rate). This can be seen as a speed-accuracy trade-off.

**Figure 5:**
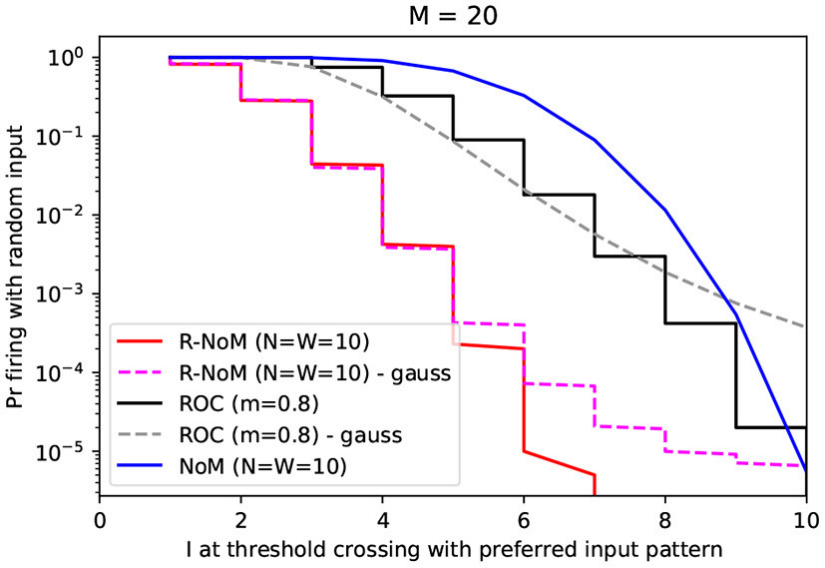
Speed-accuracy trade-off. Here, we represented, for the different codes, the probability of firing to random inputs as a function of *I* at threshold crossing for the preferred pattern (both variables depend on the threshold which is varied, but not represented on this figure). Solid lines indicate the probabilities estimated by Monte Carlo simulations (n=2.10^5^ runs for each value). Dashed lines indicate the probability assuming a Gaussian distribution for the potential, with the mean and variance computed from Table 3 formulas. These Gauss-based values match well the simulations for low latencies. For higher latencies, they tend to overestimate the FA rate. This suggests that the potential is roughly normally distributed everywhere except in the right tail of the distribution.

We explored this trade-off through numerical simulations. We fixed *M* = 20 and estimated the false alarm probabilities for ROC (*m* = 0.8), R-NoM (W=N=10), and NoM (also W=N=10), as a function of the threshold, using 2.10^5^ random input spike patterns. In Figure 5, we plotted those probabilities as a function of the latency (expressed in input spike number, not in seconds) for the firing response to the preferred pattern (latency which in turn depends on the threshold). This plot confirms the supremacy of R-NoM, especially in the early stage of the response, in agreement with Figure 4. For example, here the preferred pattern has N=10 spikes. Let's say we want the receiver neuron to fire as soon as the fifth input spike is received. For R-NoM, this means the threshold should be in the [294, 330] range. Choosing 330 will minimize the FA rate, which will be around 3.10^−4^. For ROC, the corresponding threshold would be 28.36, leading to a much higher FA rate of 0.1. Finally, for NoM, the threshold would be 5, and the FA rate 0.7, which would be totally unacceptable!

Here again, our attempt to speculate upon how to combine computation-power of float-based TTFSs schemes and power-saving integer-based TTFS schemes offers a promising avenue: FA rate could be cut by a factor of three orders of magnitude compared with the former, and four orders compared with the latter.

## 3. Discussion

In this paper, we presented a new mathematical framework which allows unifying various TTFS codes. This framework introduces the concept of modulation: a decreasing function such that the earliest input spikes matter more. This broad definition of modulation encompasses previous proposals (ROC, NoM) as well as new ones. The activation is maximal when the spikes arrive in the order of the weights: the first spike should arrive through the strongest weight, and so on. This defines the preferred input spike pattern of a neuron. Then, we defined discriminability, which measures how much more the neuron responds to its preferred pattern than to random inputs. Our framework allows us to compute this discriminability analytically. Thus various TTFS codes can easily be compared in terms of discriminability. The framework also allows the design of new codes that maximize this discriminability. In particular, we propose a new code that we dubbed “Ranked-NoM” (R-NoM), which makes use of integer modulation and weights that both decrease linearly. We demonstrated that R-NoM has much more discriminative power than ROC and NoM, especially in the early phase of the response, which is already very selective. Thus it allows detectors that are both accurate and reactive. In addition, the fact that R-NoM uses only integers makes it much more hardware-friendly than ROC, and the geometric modulation suggested in Furber et al. (2007).

There are however situations where NoM coding can be particularly interesting for hardware implementations. The advantages of R-NoM coding described here apply in situations where incoming spikes are processed one by one. However, in some designs, it is possible to process spikes as a packet. For example, you could define an input array with M bits that are initially all set to zero. As spikes come in, the corresponding input lines can be flipped on until a fixed number of bits (N) are set to one. At this point, it is easy to determine the level of activation of a target neuron by performing a logical AND operation between the array of input spikes and a second array of bits corresponding to the connected weights. Counting the number of “hits” and comparing the result to the neuron's threshold can be done in a single clock cycle with specialized FPGA or ASIC hardware. Similar results can be obtained using memristor-based cross-bar arrays.

That said, the current analysis provides a strong argument for using implementations that process incoming spikes in order since it is the only way to take advantage of the remarkable early discriminative power of R-NoM coding. Such an approach goes a long way toward ensuring that computations can be done with the minimum number of spiking events.

One important issue that we did not address in this paper is learning. We plan to address it in future work. Only then we will be able to confront the different coding schemes with real-world data (e.g., CIFAR, ImageNet, Google Speech Commands) and compare their performance, possibly using the methodology of Guo et al. (2021). For unsupervised learning, we think that the STDP-like learning rule that we proposed in Thorpe et al. (2019) could be adapted for the integer, non-binary, weights that are required for R-NoM. In short, part of the weights from unused synapses could be moved to used but not saturated synapses. For supervised learning, backpropagation has already been adapted to TTFS codes (Mostafa, 2017; Zhou et al., 2019; Kheradpisheh and Masquelier, 2020; Park et al., 2020; Sakemi et al., 2020; Zhang et al., 2020; Comsa et al., 2021; Mirsadeghi et al., 2021). Yet none of these approaches included the concept of a spike-based decreasing modulation. We will explore that possibility in future work.

## Data availability statement

The original contributions presented in the study are included in the article/Supplementary material, further inquiries can be directed to the corresponding author/s.

## Author contributions

ST and TM designed the project. LB and JG performed the mathematical derivations. JG and TM did the numerical simulations. All authors wrote the paper. All authors contributed to the article and approved the submitted version.

## Funding

The authors gratefully acknowledge financial support from the Colombian non-profit foundation COLFUTURO and the partial funding of Le Centre de Recherche Cerveau et Cognition (CerCo).

## Conflict of interest

The authors declare that the research was conducted in the absence of any commercial or financial relationships that could be construed as a potential conflict of interest.

## Publisher's note

All claims expressed in this article are solely those of the authors and do not necessarily represent those of their affiliated organizations, or those of the publisher, the editors and the reviewers. Any product that may be evaluated in this article, or claim that may be made by its manufacturer, is not guaranteed or endorsed by the publisher.
