# Supplementary material for: Analyzing time-to-first-spike coding schemes: A theoretical approach
Source: Front Neurosci. 2022 Sep 26;16:971937. doi: 10.3389/fnins.2022.971937 (PMC9548614; doi:10.3389/fnins.2022.971937)
Supplement: Supplementary file 1 [file Data_Sheet_1.pdf]

Supplementary Material

Here, we provide the full details of the theoretical mathematical framework of the temporal coding schemes. This Theoretical development is based upon multivariate calculus and probability theory. In the first section (section 1), we build the general theory by defining a random experiment in which we transform the possible input orders into a probabilized weights support. This set is then a base to build a scores support for each scheme. In this section, we define how the scores support translate into integration through modulation. As our goal is to compare the codes' discriminability power, we then establish the theory of the latter, in order to find general formulas for the maximal value of integration, its mean and variance.

Then, we apply this general theory to Ranked-NoM Coding (section 2), NoM Coding (section 3) and ROC Coding (section 4).

We provide, as another SM, a Jupyter-Notebook that numerically validates all results.

1 GENERAL FRAMEWORK

1.1 General principles

Let us consider an input layer made of M spiking neurones, denoted $I_0, .., I_{(M-1)}$, which send spikes to an efferent neurone.

For a given stationary stimulus, each of the M input neurones emits one spike, and the order in which spikes are emitted depends on the stimulus. We encode the order of the input spikes by the vector corresponding to the order of neurones' indices. For instance, the order $(I_0, .., I_{(M-1)})$ will translate into $(0, 1, \dots, M-1)$, and the inverse order will translate into $(M-1, M-2, \dots, 1, 0)$.

This input order is transformed into a vector of weights.

Depending on the scheme, the vector of weights is used to build a vector of scores.

The response of an efferent neurone is then built as a sum of the scores multiplied by a modulation which is specific for each input neurone.

1.2 Random Experiment

We consider the order generated by the M input neurones as a random sequence, where all input orders have the same probability.

1.2.1 Input orders

Let Λ denote the ascending lexically ordered set of the possible permutations over the set $\mathcal{M} = \{0, \dots, M-1\}$: $\Lambda = \{\mathbf{r}^1, ..., \mathbf{r}^{M!}\}$ where $\mathbf{r} \in \mathbb{R}^M$ and $r_i^j \in \mathcal{M}$. To map a rank into an input order in Λ, we define the application $\mathcal{R} : \{1, 2, .., M!\} \to \Lambda$ such that $\mathcal{R}(k) = \mathbf{r}^k$.

We consider the discrete random variable K, defined as:

$$K = \begin{cases} \mathcal{D}_K = \{1, 2, .., M!\} \\ \mathrm{P}_K(k) = \frac{1}{M!} \end{cases}$$

Building upon K, we can then consider $\mathbf{X} = \mathcal{R}(K) = (X_1, X_2, \ldots, X_M)$ [1] as a random vector or multivariate random variable with support the ordered set Λ:

$$\Lambda_{X_1 X_2 \ldots X_M} = \left\{ \begin{bmatrix} 0 \\ 1 \\ \vdots \\ M-1 \end{bmatrix} \begin{bmatrix} 1 \\ 0 \\ \vdots \\ M-1 \end{bmatrix} \cdots \begin{bmatrix} M-1 \\ \vdots \\ 1 \\ 0 \end{bmatrix} \right\} \tag{1.1}$$

By construction, it is a vector of size M, and each component X_i is a random variable defined on the same sample space obeying:

$$X_i = \begin{cases} \mathcal{D}_{X_i} = \{0, \ldots, M-1\} \\ \\ \mathrm{P}_{X_i}(r_i) = \frac{1}{M} \end{cases}$$

Let its multivariate joint probability mass function be

$$P_{X_1 X_2 \ldots X_M} = \begin{cases} \dfrac{1}{M!} & \mathbf{r}^k \in \Lambda_{X_1 X_2 \ldots X_M} \\ 0 & \text{Otherwise} \end{cases} \tag{1.2}$$

$X_1, X_2, \ldots, X_M$ are identically distributed, hence they have the same expectation and variance; they are not independent given that:

$$P_{X_1}(r) P_{X_2}(r) \ldots P_{X_M}(r) = \left(\frac{1}{M}\right)^M \neq \frac{1}{M!} = P_{X_1 X_2 \ldots X_M} \tag{1.3}$$

thus $\mathrm{Cov}(X_i, X_j) \neq 0$.

1.2.2 From input order to weights

We define the affine transformation Φ mapping vectors from Λ (input orders) to the set Ω (weights):

$$\begin{aligned} \Phi: \quad \Lambda \subset \mathbb{R}^M &\longrightarrow \quad \Omega \subset \mathbb{R}^M \\ (r_1^k, \ldots, r_M^k) &\longrightarrow (\hat{w}_1^k, \ldots, \hat{w}_M^k) \\ \mathbf{r}^k &\longrightarrow \quad \hat{\mathbf{w}}^k \end{aligned} \tag{1.4}$$

Each vectorial component is given by

$$\phi_i(\mathbf{r}^k) = \phi_i(r_1^k, \ldots, r_M^k) = M - r_i^k = \hat{w}_i^k \tag{1.5}$$

so we have:

$$\begin{aligned} \Phi(\mathbf{r}^k) &= \Phi(r_1^k, \ldots, r_M^k) && (1.6) \\ &= (\phi_1(r_1^k, \ldots, r_M^k), \phi_2(r_1^k, \ldots, r_M^k), \ldots, \phi_M(r_1^k, \ldots, r_M^k)) && (1.7) \\ &= (M - r_1^k, M - r_2^k, \ldots, M - r_M^k) && (1.8) \\ &= (\hat{w}_1^k, \ldots, \hat{w}_M^k) = \hat{\mathbf{w}}^k && (1.9) \end{aligned}$$

Consider $X_1, ..., X_M$, a set of random variables with joint probability distribution function $P_{X_1 \ldots X_M}(x_1, \ldots, x_M)$ and support Λ. Let us denote $\hat{W}_i^k = \phi_i(X_1, ..., X_M)$ for $i = 1, \ldots, M$. We

[1] Throughout the article, we will use rows or columns interchangeably to denote random variables and define vector-value functions.

transform the random variables X_i (input order) into the new random variables $\hat{W}_i$ (weights) by the affine transformation Φ.

Since the multivariate transformation Φ is one to one, the transformation is invertible and can be solved for the equations $r_i^k = \phi_i^{-1}(\hat{w}_1^k, \ldots, \hat{w}_M^k)$ for $i = 1, \ldots, M$.

The Jacobian of this multivariate transformation is

$$J\Phi = \begin{vmatrix} \nabla\phi_1 \\ \nabla\phi_2 \\ \vdots \\ \nabla\phi_M \end{vmatrix} = \begin{vmatrix} \frac{\partial\phi_1}{\partial r_1^k} & \frac{\partial\phi_1}{\partial r_2^k} & \cdots & \frac{\partial\phi_1}{\partial r_M^k} \\ \frac{\partial\phi_2}{\partial r_1^k} & \frac{\partial\phi_2}{\partial r_2^k} & \cdots & \frac{\partial\phi_2}{\partial r_M^k} \\ \vdots & \vdots & \ddots & \vdots \\ \frac{\partial\phi_M}{\partial r_1^k} & \frac{\partial\phi_M}{\partial r_2^k} & \cdots & \frac{\partial\phi_M}{\partial r_M^k} \end{vmatrix} = \begin{vmatrix} -1 & 0 & \ldots & 0 \\ 0 & -1 & \ldots & 0 \\ \vdots & \vdots & \ddots & \vdots \\ 0 & 0 & \ldots & -1 \end{vmatrix} = -1 \tag{1.10}$$

Let $|J\Phi|$ denote the absolute value of the determinant J . Then the joint pdf of $\hat{W}_1^k, \ldots, \hat{W}_M^k$ is

$$P_{\hat{W}_1 \ldots \hat{W}_M}(\hat{w}_1^k, \ldots, \hat{w}_M^k) = P_{X_1 \ldots X_M}(\phi_1^{-1}(\hat{w}_1^k), \ldots, \phi_M^{-1}(\hat{w}_1^k))|J\Phi| \tag{1.11}$$

The inverse for $i = 1, \ldots, M$ is given by

$$\phi_i^{-1} = M - w_i^k = r_i^k \tag{1.12}$$

Therefore, $\hat{\mathbf{W}} = (\hat{W}_1, \hat{W}_2 \ldots, \hat{W}_M)$ is a discrete random vector with weights-support the ordered set $\Omega = \{\hat{\mathbf{w}}_1^k, \ldots, \hat{\mathbf{w}}_{M!}^k\}$

$$\Omega_{\hat{W}_1 \hat{W}_2 \ldots \hat{W}_M} = \left\{ \begin{bmatrix} M \\ M-1 \\ \vdots \\ 1 \end{bmatrix} \begin{bmatrix} M-1 \\ M \\ \vdots \\ 1 \end{bmatrix} \cdots \begin{bmatrix} 1 \\ \vdots \\ M-1 \\ M \end{bmatrix} \right\} \tag{1.13}$$

and its joint pdf is given by

$$\begin{aligned} P_{\hat{W}_1 \ldots \hat{W}_M}(\hat{w}_1^k, \ldots, \hat{w}_M^k) &= P_{X_1 \ldots X_M}(\phi_1^{-1}(\hat{w}_1^k), \ldots, \phi_M^{-1}(\hat{w}_1^k))|J\Phi| \\ &= P_{X_1 \ldots X_M}(\phi_1^{-1}(\hat{w}_1^k), \ldots, \phi_M^{-1}(\hat{w}_1^k)) \\ &= P_{X_1 \ldots X_M}(M - w_1^k, \ldots, M - w_M^k) \\ &= \frac{1}{M!} \end{aligned} \tag{1.14}$$

In order to find the *marginal probability mass function* of $\hat{W}_1, \hat{W}_2, \ldots, \hat{W}_M$, we start from the joint probability distribution of $\hat{W}_i$, and proceed by summation (see Fig. S1).

Thus, the *marginal probability mass function* of $\hat{W}_1, \hat{W}_2, \ldots, \hat{W}_M$ defined on the same sample space $\mathcal{D}_{X_i} = \{M, M-1, ..., 1\}$ for $i = 1, ..., M$, with joint probability distribution defined in the equation (1.14), and for equation (1.17) is given by,

$$P_{\hat{W}_i}(w) = \begin{cases} \frac{(M-1)!}{M!} = \frac{1}{M} & w = 1, 2, \ldots M \\ 0 & \text{Otherwise} \end{cases} \tag{1.18}$$

Let $Y_1, Y_2, \ldots, Y_n$ be discrete random variables defined on the same sample space $\mathcal{D}_{Y_j} = \{y_1, y_2, \ldots, y_m\}$ and $P_{Y_1 Y_2 \ldots Y_n}$ be the multivariate probability distribution of the random variables Y_j, then we have:

$$P_{Y_1}(y_1) = \sum_{y_{i_n} \in \mathcal{D}_{Y_n}} \cdots \sum_{y_{i_2} \in \mathcal{D}_{Y_2}} P_{Y_1 Y_2 \ldots Y_n}(y_1, y_{i_2}, \ldots, y_{i_n}) \tag{1.15}$$

$$\vdots \qquad\qquad\qquad\qquad \vdots$$

$$P_{Y_1}(y_m) = \sum_{y_{i_n} \in \mathcal{D}_{Y_n}} \cdots \sum_{y_{i_2} \in \mathcal{D}_{Y_2}} P_{Y_1 Y_2 \ldots Y_n}(y_m, y_{i_2}, \ldots, y_{i_n}) \tag{1.16}$$

and the same way, we can compute the probability of each element of the sample space of $Y_2, \ldots, Y_n$. The marginal probability function of βth random variable Y_β of γth output y_γ, $P_{Y_\beta}(y_\gamma)$, is given by:

$$P_{Y_\beta}(y_\gamma) = \sum_{y_{i_n} \in \mathcal{D}_{Y_n}} \cdots \sum_{\substack{y_{i_k} \in \mathcal{D}_{Y_k} \\ k \neq \beta}} \cdots \sum_{y_{i_1} \in \mathcal{D}_{Y_1}} P_{Y_1 \ldots Y_\beta \ldots Y_n}(y_{i_1}, \ldots, y_\gamma, \ldots, y_{i_n}) \tag{1.17}$$

Figure S1. Marginal probability mass function from joint probability distribution

1.3 Coding theory-definitions

A coding scheme is defined by how weights are transformed to scores, and how these scores are integrated with a modulation function.

1.3.1 From weights to scores

To attribute scores to the weights for a given coding scheme, we define the *score vector*, $\mathbf{w}_C^k = (w_1^k, \ldots, w_M^k) \in \mathbb{R}^M$, by the following function Φ_C:

Definition 1.1. We define the vector-valued function,

$$\begin{aligned} \Phi_C: \quad \Omega \subset \mathbb{R}^M \quad &\longrightarrow \quad \Omega_C \subset \mathbb{R}^M \\ (\hat{w}_1^k, \ldots, \hat{w}_M^k) &\longrightarrow (w_1^l, \ldots, w_M^l) \\ \hat{\mathbf{w}}^k \quad &\longrightarrow \quad \mathbf{w}_C^l \end{aligned} \tag{1.19}$$

such that:

$$\Phi_C(\hat{\mathbf{w}}^k) = \Phi_C(\hat{w}_1^k, \ldots, \hat{w}_M^k) \tag{1.20}$$

$$= (\phi_{C,1}(\hat{w}_1^k, \ldots, \hat{w}_M^k), \ldots, \phi_{C,M}(\hat{w}_1^k, \ldots, \hat{w}_M^k)) \tag{1.21}$$

$$= (w_1^l, \ldots, w_M^l) = \mathbf{w}_C^l \tag{1.22}$$

where $l \in \{1, \ldots, |\Omega_C|\}$ and $\phi_{C,i}$ are real functions of several variables, $\phi_{C,i} : \Omega \to \mathbb{R}$ that are defined depending on the coding scheme.

Therefore, let $\mathbf{W} = (W_1, W_2 \ldots, W_M)$ be a discrete random vector with support the ordered set $\Omega_C = \{\mathbf{w}_1^l, \ldots, \mathbf{w}_{|\Omega_C|}^l\}$ where $\mathbf{w}_C^l$ are the scores of scheme C.

Being built upon $\mathbf{W}$, the components W_i of $\mathbf{W}$ are also identically distributed hence they have the same expectation and variance. We will show below they are not independent, and will establish their covariance by *tree method*, one for each scheme (2.32)(3.30)(4.26).

We denote Ω_C the set of possible values for $\mathbf{w}_C^k$, that is, the scores-support and its cardinality $|\Omega_C|$ depends on the coding scheme.

1.3.2 Modulations vector function

In order to sort out among stimuli by output neurones, vectors of modulation values must be defined with the same size as $\mathbf{w}_C^k$. In the same way as we defined $\mathbf{w}^k$, modulation vectors $\mathbf{v}^l$ can be defined by a modulation function following:

Definition 1.2. We define the application,

$$\begin{aligned} \Psi_C: \quad \Omega \subset \mathbb{R}^M \quad &\longrightarrow \quad \Xi_C \subset \mathbb{R}^M \\ (\hat{w}_1^k, \ldots, \hat{w}_M^k) &\longrightarrow (v_1^l, \ldots, v_M^l) \\ \hat{\mathbf{w}}^k \quad &\longrightarrow \quad \mathbf{v}_C^l \end{aligned} \tag{1.23}$$

such that:

$$\Psi_C(\hat{\mathbf{w}}^k) = \Psi_C(\hat{w}_1^k, \ldots, \hat{w}_M^k) \tag{1.24}$$

$$= (\psi_{C,1}(\hat{w}_1^k, \ldots, \hat{w}_M^k), \ldots, \psi_{C,M}(\hat{w}_1^k, \ldots, \hat{w}_M^k)) \tag{1.25}$$

$$= (v_1^l, \ldots, v_M^l) = \mathbf{v}_C^l \tag{1.26}$$

where $l \in \{1, \ldots, |\Xi|\}$ and $\psi_{C,i}$ are real functions of several variables, $\psi_{C,i} : \Omega \to \mathbb{R}$ that depend upon the coding scheme.

We denote Ξ_C the set of possible values for $\mathbf{v}_C^k$ and its cardinality depends on the coding scheme through Ψ_C.

The modulation vector determines which input order is preferred by the efferent neurone under consideration. For the sake of clarity, we will consider from now on only the output neurone for which the preferred stimulus (input order) is the one corresponding to $\mathbf{r}^1$. Hence, we will use

$$\mathbf{v}_C^1 = \Psi_C(\hat{\mathbf{w}}^1) = \Psi_C(\Phi(\mathbf{r}^1)) \tag{1.27}$$

as the modulation vector.

1.3.3 Integration function $S_C(\mathbf{w}_C^k, I)$

To compare how *scores vector* matches well with *modulations vector*, we define an *integration function* following:

Definition 1.3. We define the application $S_C(\mathbf{w}_C^k, I) : \Omega_C \times \{0, \ldots, M\} \to \mathbb{R}$ such as:

$S_C(\mathbf{w}_C^k, I)$ is the inner product of *modulations vector* $\mathbf{v}_C^1$ (set for best matching $\hat{\mathbf{w}}^1$ for the coding scheme C) by *score vector* $\mathbf{w}_C^k$, over the I first components of vectors.

In order to formally translate intermediate states, we first define the gate function $G_I : \Xi_C \to \mathbb{R}^M$ which nullifies all components of the modulation vector for ranks beyond I.

Then, the integration function reads:

$$S_C(\mathbf{w}_C^k, I) = \left\langle G_I\left(\Psi_C(\hat{\mathbf{w}}^1)\right), \mathbf{w}_C^k \right\rangle \tag{1.28}$$

where $G_I\left(\Psi_C(\hat{\mathbf{w}}^1)\right) = G_I\left(\mathbf{v}_C^1\right) = \mathbf{v}_{C,I}^1$. We used bracket notation for inner product.

Building upon the random variable $\mathbf{W}$, we finally define the random variable $S_{C,I} = S_C(\mathbf{W}, I)$:

$$S_{C,I} = \begin{cases} \mathcal{D}_{S_{C,I}} = \{\textit{depends upon } C\} \\ \\ \mathrm{P}_{S_{C,I}} = \{\textit{depends upon } C\} \end{cases}$$

We can then define the best order $\max(S_{C,I})$, expectation $\mathrm{E}[S_{C,I}]$ and variance $\mathrm{Var}[S_{C,I}]$ for each coding scheme.

1.4 Discriminability power measure

The goal is to compare the coding schemes by their power to discriminate among stimuli.

1.4.1 Discriminability

Considering integration $S_{C,I}$ random variable, we define *discriminability* $D_C(I)$ as the difference between its best possible value and its expectation, scaled by its variance :

Definition 1.4.

$$D_C(I) = \frac{\max(S_{C,I}) - \mathrm{E}[S_{C,I}]}{\sqrt{\mathrm{Var}[S_{C,I}]}}, I \in \{1, \ldots, M\} \tag{1.29}$$

where $I \in \{1, \ldots, M\}$ for ROC and $I \in \{1, \ldots, \mathcal{N}\}$ for Ranked-NoM and NoM coding. Given that the Ranked-NoM and NoM schemes are not defined for $\mathcal{N} < I < M$, we use the final integration value.

To compute discriminability for the different coding schemes, we then now turn to the expressions of $\max(S_{C,I})$, $\mathrm{E}[S_{C,I}]$ and $\mathrm{Var}[S_{C,I}]$.

1.4.2 max($S_{C,I}$)

The rearrangement inequality states that

$$\begin{pmatrix} x_n \\ \vdots \\ x_1 \end{pmatrix}^\mathsf{T} \cdot \begin{pmatrix} y_1 \\ \vdots \\ y_n \end{pmatrix} \leq \begin{pmatrix} x_{\sigma(1)} \\ \vdots \\ x_{\sigma(n)} \end{pmatrix}^\mathsf{T} \cdot \begin{pmatrix} y_1 \\ \vdots \\ y_n \end{pmatrix} \leq \begin{pmatrix} x_1 \\ \vdots \\ x_n \end{pmatrix}^\mathsf{T} \cdot \begin{pmatrix} y_1 \\ \vdots \\ y_n \end{pmatrix} \tag{1.30}$$

for every choice of real numbers $x_1 \leq \cdots \leq x_n$ and $y_1 \leq \cdots \leq y_n$ and every permutation $x_{\sigma(1)}, \ldots, x_{\sigma(n)}$ of $x_1, \ldots, x_n$.

Then the lower bound is attained only for the permutation which reverses the order, that is, $\sigma(i) = n - i + 1$ for all $i = 1, \ldots, n$, and the upper bound is attained only for the identity, that is, $\sigma(i) = i$.

From equation (1.28), the rearrangement inequality yields that $\max(S_{C,I})$ is given by:

$$\max(S_{C,I}) =< \mathbf{v}^1_{C,I}, \mathbf{w}^1_C > \tag{1.31}$$

1.4.3 Expectation of $S_{C,I}$

For the sake of clarity, let $\mathbf{v}_{C,I} = \mathbf{v}^1_{C,I} = G_I\left(\Psi_C(\mathbf{r}^1)\right)$ denote the modulation vector, gated up to the first I components.

We have:

$$\begin{aligned} \mathrm{E}[S_{C,I}] &= \mathrm{E}[S_C(\mathbf{W}, I)] \\ &= \mathrm{E}\left[\langle \mathbf{v}_{C,I}, \mathbf{W}\rangle\right] \\ &= \mathbf{v}^\mathsf{T}_{C,I} \cdot \mathrm{E}\left[\mathbf{W}\right] \end{aligned} \tag{1.32}$$

where the expected value of a random vector is the vector whose elements are the expected values of the respective random variables.

1.4.4 Variance of $S_{C,I}$

Using the same notation $\mathbf{v}_{C,I}$, the variance of $S_C(K, I)$ reads:

$$\mathrm{Var}\left[S_{C,I}\right] = \mathrm{Var}\left[S_C(\mathbf{W}, I)\right] \tag{1.33}$$

$$= \mathrm{Var}\left[\mathbf{v}^\mathsf{T}_{C,I} \cdot \mathbf{W}\right] \tag{1.34}$$

$$= \mathrm{E}[\mathbf{v}^\mathsf{T}_{C,I} \cdot \mathbf{W} \cdot \mathbf{W}^\mathsf{T} \cdot \mathbf{v}_{C,I}] - \mathrm{E}[\mathbf{v}^\mathsf{T}_{C,I} \cdot \mathbf{W}] \cdot \mathrm{E}[\mathbf{v}^\mathsf{T}_{C,I} \cdot \mathbf{W}]^\mathsf{T} \tag{1.35}$$

$$= \mathbf{v}^\mathsf{T}_{C,I} \cdot \mathrm{E}[\mathbf{W} \cdot \mathbf{X}^\mathsf{T}] \cdot \mathbf{v}_{C,I} - \mathbf{v}^\mathsf{T}_{C,I} \cdot \mathrm{E}[\mathbf{W}] \cdot \mathrm{E}[\mathbf{W}]^\mathsf{T} \cdot \mathbf{v}_{C,I} \tag{1.36}$$

$$= \mathbf{v}^\mathsf{T}_{C,I} \cdot (\mathrm{E}[\mathbf{W} \cdot \mathbf{W}^\mathsf{T}] - \mathrm{E}[\mathbf{W}] \cdot \mathrm{E}[\mathbf{W}]^\mathsf{T}) \cdot \mathbf{v}_{C,I} \tag{1.37}$$

$$= \mathbf{v}^\mathsf{T}_{C,I} \cdot \mathbf{K}_{WW} \cdot \mathbf{v}_{C,I} \tag{1.38}$$

where $\mathbf{K}_{WW}$ is the $M \times M$ Variance-Covariance Matrix of $\mathbf{W}$:

$$\mathbf{K}_{WW} = \mathrm{E}[(\mathbf{W} - \mathrm{E}[\mathbf{W}])(\mathbf{W} - \mathrm{E}[\mathbf{W}])^{\mathsf{T}}] \tag{1.39}$$
$$= \mathrm{E}[\mathbf{W}\ \mathbf{W}^{\mathsf{T}}] - \mathrm{E}[\mathbf{W}]\ \mathrm{E}[\mathbf{W}]^{\mathsf{T}}$$
$$= \begin{bmatrix} \mathrm{Cov}(W_1, W_1) & \mathrm{Cov}(W_1, W_2) & \dots & \mathrm{Cov}(W_1, W_M) \\ \mathrm{Cov}(W_2, W_1) & \mathrm{Cov}(W_2, W_2) & \dots & \mathrm{Cov}(W_2, W_M) \\ \vdots & \vdots & \ddots & \vdots \\ \mathrm{Cov}(W_M, W_1) & \mathrm{Cov}(W_M, W_2) & \dots & \mathrm{Cov}(W_M, W_M) \end{bmatrix} \tag{1.40}$$

where covariance $\mathrm{Cov}(W_i, W_j)$ is defined as:

$$\mathrm{Cov}(W_i, W_j) = \mathrm{E}[(W_i - \mathrm{E}[W_i])(W_j - \mathrm{E}[W_j])] \tag{1.41}$$
$$= \sum_{i,j}(w_i - \mu_W)(w_j - \mu_W)f(W_i = w_i, W_j = w_j) \tag{1.42}$$

where $\mu_W = \mathrm{E}[W_i] = \mathrm{E}[W_j]$ and $f(W_i, W_j)$ is the bivariate joint probability distribution.

The diagonal elements are the variance of W_i with $\mathrm{Cov}(W_i, W_i) = \mathrm{E}[(W_i - \mathrm{E}[W_i])^2] = \mathrm{Var}(W_i)$.

Since $\mathbf{W}$ elements are identically distributed, they all have the same variance and the same co-variance, and $\mathrm{Cov}(W_i, W_j) = \mathrm{Cov}(W_j, W_i)$.

$\mathbf{K}_{WW}$ is then symmetric and has only two values, an on-diagonal value $\mathrm{Var}(W_i) \equiv \mathrm{Var}W$, and an off-diagonal value $\mathrm{Cov}(W_i, W_j) \equiv \vartheta$, reading:

$$\mathbf{K}_{WW} = \begin{bmatrix} \mathrm{Var}W & \vartheta & \dots & \vartheta \\ \vartheta & \mathrm{Var}W & \dots & \vartheta \\ \vdots & \vdots & \ddots & \vdots \\ \vartheta & \vartheta & \dots & \mathrm{Var}W \end{bmatrix} \tag{1.43}$$

We denote the variance-covariance matrix for each C scheme as $\mathbf{K}^C_{WW}$.

2 APPLICATION TO RANKED-NOM CODING

2.1 Score vector function

Ranked-NoM Coding is parameterized by the number $\mathcal{W} \in \{1, \dots, M\}$. Under this coding, we want the best weight vector $\mathbf{w}^1$ made up of an arithmetic sequence, over the first $\mathcal{W}$ elements, starting from $\mathcal{W}$, with rate -1, down to zero, and the other elements set to zero.

The scores are obtained by the vector-value function $\Phi_H(\hat{\mathbf{w}}^k) = \mathbf{w}^l_H$ defined in (1.1). Then the vectorial components are given by,

$$\phi_{H,i}(\hat{\mathbf{w}}^k) = \phi_{H,i}(\hat{w}^k_1, \dots, \hat{w}^k_M) = \max(0, \hat{w}^k_i - M + \mathcal{W}) \tag{2.1}$$

such that:

$$\Phi_H(\hat{\mathbf{w}}^k) = \Phi_H(\hat{w}^k_1, \dots, \hat{w}^k_M) \tag{2.2}$$
$$= \Phi_H(M - r^k_1, M - r^k_2, \dots, M - r^k_M) \tag{2.3}$$
$$= (\phi_{H,1}(\hat{w}^k_1, \dots, \hat{w}^k_M), \dots, \phi_{H,M}(\hat{w}^k_1, \dots, \hat{w}^k_M)) \tag{2.4}$$
$$= (\max(0, \mathcal{W} - r^k_1), \dots, \max(0, \mathcal{W} - r^k_M))$$
$$= (w^l_1, \dots, w^l_M) = \mathbf{w}^l_H \tag{2.5}$$

Note that for $k = 1$, we have $\hat{\mathbf{w}}^1 = (M, M-1\ldots, 1)$ or equivalently $\mathbf{r}_i^1 = (0, \ldots, M-1)$

$$\Phi_H(\hat{\mathbf{w}}^1) = (\max(0, \mathcal{W}), \ldots, \max(0, \mathcal{W} - M + 1)) \tag{2.6}$$
$$= (\mathcal{W}, \mathcal{W} - 1, \ldots, 0) = \mathbf{w}_H^1 \tag{2.7}$$

which contains $M - \mathcal{W}$ zeros.

Example 2.1. As an illustration, let $M = 4$ and $\mathcal{W} = 2$ ($|\Omega| = 24$ permutations). We would have, for the best order:

$$\Phi_H(\hat{\mathbf{w}}^1) = (\max(0, 2), \max(0, 1), \max(0, 0), \max(0, -1)) \tag{2.8}$$
$$= (2, 1, 0, 0) = \mathbf{w}_H^1 \tag{2.9}$$

and for the worst order:

$$\Phi_H(\hat{\mathbf{w}}^{24}) = (\max(0, -1), \max(0, 0), \max(0, 1), \max(0, 2)) \tag{2.10}$$
$$= (0, 0, 1, 2) \tag{2.11}$$

Note that for $\hat{\mathbf{w}}^{18} = (2, 1, 3, 4)$, we would also obtain:

$$\Phi_H(\hat{\mathbf{w}}^{18}) = (\max(0, 0), \max(0, -1), \max(0, 1), \max(0, 2)) \tag{2.12}$$
$$= (0, 0, 1, 2) \tag{2.13}$$

therefore, $\Phi_H(\mathbf{w}^{18}) = \Phi_H(\hat{\mathbf{w}}^{24})$.

Indeed, Φ_H maps different weights permutations onto the same score permutation. Hence, a subset of vectors that are pure internal permutations of negative or null values will map to the same element of Ω_H. Since the cardinality of these subsets is the number of permutations of the $M - \mathcal{W}$ null elements, the cardinality of Ω_H is:

$$|\Omega_H| = \frac{M!}{(M - \mathcal{W})!} \tag{2.14}$$

In the illustrative example, $|\Omega_H| = \dfrac{4!}{(4-2)!} = 12$ permutations, and we would have $\Phi_H(\hat{\mathbf{w}}^{18}) = \Phi_H(\hat{\mathbf{w}}^{24}) = \mathbf{w}^{12}$.

2.2 Probability distribution functions for scores

Let $\mathbf{W} = (W_1, W_2, \ldots, W_M)$ be the discrete random vector with support the ordered set $\Omega_H = \{\mathbf{w}^1, \ldots, \mathbf{w}^{\frac{M!}{(M-\mathcal{W})!}}\}$ which is generated by function $\hat{\Phi}_H$ defined in 2.1, and :

$$\Omega_H = \Omega_{W_1 W_2 \ldots W_M} = \left\{ \begin{bmatrix} \mathcal{W} \\ \mathcal{W}-1 \\ \vdots \\ 1 \\ 0 \\ \vdots \\ 0 \end{bmatrix} \begin{bmatrix} \mathcal{W}-1 \\ \mathcal{W} \\ \vdots \\ 1 \\ 0 \\ \vdots \\ 0 \end{bmatrix} \ldots \begin{bmatrix} 0 \\ \vdots \\ 0 \\ 1 \\ \vdots \\ \mathcal{W}-1 \\ \mathcal{W} \end{bmatrix} \right\} \tag{2.15}$$

Due to (2.14) let its *joint probability mass function* be

$$P_{W_1W_2...W_M} = \begin{cases} \dfrac{1}{|\Omega_H|} = \dfrac{(M-\mathcal{W})!}{M!} & \mathbf{w}^k \in \Omega_H \\ 0 & \text{Otherwise} \end{cases} \tag{2.16}$$

and from (1.17), the *marginal probability mass function* of $W_1, W_2, \dots, W_M$ with sample space $\mathcal{D}_{W_i} = \{\mathcal{W}, \mathcal{W}-1, ..., 1, 0\}$ for $i = 1, ..., M$, is given by:

$$P_{W_i}(w=0) = \frac{M-\mathcal{W}}{M} \tag{2.17}$$

$$P_{W_i}(w=k) = \frac{1}{M} \tag{2.18}$$

where $k = 1, ..., \mathcal{W}-1, \mathcal{W}$.

Therefore,

$$W_i = \begin{cases} \mathcal{D}_{W_i} = \{0, 1, ..., \mathcal{W}\} \\ \mathrm{P}_{W_i}(w) = \begin{cases} \dfrac{M-\mathcal{W}}{M} & w = 0 \\ \dfrac{1}{M} & w = 1, 2, \dots \mathcal{W} \end{cases} \end{cases} \tag{2.19}$$

2.3 Expectation vector of scores

2.3.1 $\boldsymbol{\mu_W}$, expected value of $\boldsymbol{W_i}$

$$\mu_W \equiv \mathrm{E}[W_i] = \sum_{k=0}^{M} k\ P_{W_i}(k) = 0\frac{M-\mathcal{W}}{M} + \sum_{k=1}^{\mathcal{W}} k\ \frac{1}{M} \tag{2.20}$$

$$= \frac{1}{M}\sum_{k=1}^{\mathcal{W}} k$$

$$= \frac{1}{M}\frac{\mathcal{W}(\mathcal{W}+1)}{2} \tag{2.21}$$

2.3.2 $\mathrm{E}[\mathbf{W}]$, expectation of $\mathbf{W}$

Therefore,

$$\mathrm{E}[\mathbf{W}] = \begin{bmatrix} E[W_1] \\ E[W_2] \\ \vdots \\ E[W_M] \end{bmatrix} = \begin{bmatrix} \dfrac{\mathcal{W}(\mathcal{W}+1)}{2M} \\ \vdots \\ \dfrac{\mathcal{W}(\mathcal{W}+1)}{2M} \end{bmatrix} \tag{2.22}$$

2.4 Variance-Covariance matrix of scores

The matrix $\mathbf{K}_{WW}^{H}$ is defined in (1.43) then we will find the variance $\mathrm{Var}W$ and the covariance $\mathrm{Cov}(W_i, W_j) = \vartheta$.

2.4.1 Var$\boldsymbol{W}$, variance of $\boldsymbol{W_i}$

Following 2.19, we have:

$$\mathrm{E}[W_i^2] = \sum_{k=0}^{\mathcal{W}} k^2 \, P_{W_i}(k) = 0^2 \frac{M-\mathcal{W}}{M} + \sum_{k=1}^{\mathcal{W}} k^2 \frac{1}{M} \tag{2.23}$$

$$= \frac{1}{M} \sum_{i=1}^{\mathcal{W}} k^2$$

$$= \frac{1}{M} \frac{\mathcal{W}(\mathcal{W}+1)(2\mathcal{W}+1)}{6} \tag{2.24}$$

Therefore,

$$\mathrm{Var}[W_i] = \mathrm{E}[W_i^2] - \mathrm{E}[W_i]^2 \tag{2.25}$$

$$= \frac{1}{M} \frac{\mathcal{W}(\mathcal{W}+1)(2\mathcal{W}+1)}{6} - \left(\frac{1}{M} \frac{\mathcal{W}(\mathcal{W}+1)}{2} \right)^2 \tag{2.26}$$

$$= \mu_W \frac{(2\mathcal{W}+1)}{3} - \mu_W^2 \tag{2.27}$$

2.4.2 $\boldsymbol{\vartheta}$, covariance of $\boldsymbol{W_i}, \boldsymbol{W_j}$

From equations(2.16) and (2.19), the score random variables W_i of Ranked-NoM coding are not independent because:

$$\prod_{i=1}^{M} P_{W_i}(w) = \left(\frac{M-N}{M} \right)^{M-N} \left(\frac{1}{M} \right)^{N} \neq \frac{(M-N)!}{M!} = P_{W_1 W_2 \ldots W_M} \tag{2.28}$$

To find the covariance (1.42), we must find the bivariate joint probability distribution $f(W_i = w_i, W_j = w_j)$, for $i, j = 1, 2, \ldots, M$.

We will use the following facts:

1. Each random variable W_i can take on $\mathcal{W}$ same values: $0, 1, 2, \ldots, \mathcal{W}$.
2. The bivariate joint probability distribution $f(W_i, W_j)$ is the same for any $i, j = 1, \ldots, M$.
3. $f(W_i = k, W_j = k) = 0$ for $k \in \{1, 2, \ldots \mathcal{W}\}$
4. The support Ω_H, which is the sample space, consists of $|\Omega_H| = \dfrac{M!}{(M-\mathcal{W})!}$ possible pair outcomes that are equally likely, that is, the probability for each random outcome is $\dfrac{1}{|\Omega_H|}$.

From fact (2), we only need to establish $f(W_1, W_2)$. From fact (3), we only need to establish the outcome frequency for each pair in:

$$(\mathcal{W}, \mathcal{W}-1), (\mathcal{W}, \mathcal{W}-2), \ldots, (\mathcal{W}-1, \mathcal{W}), (\mathcal{W}-1, \mathcal{W}-2), \ldots, (0, 0).$$

We first note that there are only three possibilities for the outcome frequencies, that is, for the pairs: $(0, 0), (0, y), (x, y)$ where $x, y \in \{\mathcal{W}, \mathcal{W}-1, \mathcal{W}-2, \ldots, 1\}$ can take any non-zero integer value. We denote :

$$(0,0)_f = f_1 \tag{2.29}$$

$$(0,y)_f = (y,0)_f = f_2 \tag{2.30}$$

$$(x,y)_f = (y,x)_f = f_3 \tag{2.31}$$

We can then enumerate the $|\Omega_H|$ possible outcomes of $\mathbf{W}$ within a tree, with the first level corresponding to values taken by W_1, and the second level corresponding to values taken by W_2 once W_1 is fixed, with leaves indicating the number of times each pair repeats:

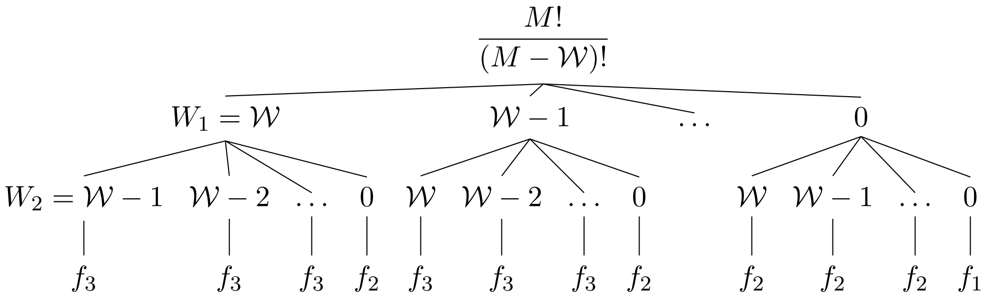

Tree for Ranked-NoM code (2.32)

For each branch, once values are given to W_1 and W_2, there are $(M-2)!$ permutations to consider, among which permutations corresponding to switching two null values should be considered the same. For a given branch, this number of permutations switching null values depends on the number of null already attributed to W_1 or W_2, hence:

$$f_1 = \frac{(M-2)!}{(M-\mathcal{W}-2)!} \tag{2.33}$$

$$f_2 = \frac{(M-2)!}{(M-\mathcal{W}-1)!} \tag{2.34}$$

$$f_3 = \frac{(M-2)!}{(M-\mathcal{W}-0)!} \tag{2.35}$$

Considering (from fact (4)) that the total number of possible pairs outcomes is $|\Omega_H| = \dfrac{M!}{(M-\mathcal{W})!}$, the corresponding probabilities are given by:

$$P_1 = \frac{f_1}{\frac{M!}{(M-\mathcal{W})!}} = \frac{\frac{(M-2)!}{(M-\mathcal{W}-2)!}}{\frac{M!}{(M-\mathcal{W})!}} = \frac{(M-\mathcal{W}-1)(M-\mathcal{W})}{(M-1)M} \tag{2.36}$$

$$P_2 = \frac{f_2}{\frac{M!}{(M-\mathcal{W})!}} = \frac{\frac{(M-2)!}{(M-\mathcal{W}-1)!}}{\frac{M!}{(M-\mathcal{W})!}} = \frac{M-\mathcal{W}}{(M-1)M} \tag{2.37}$$

$$P_3 = \frac{f_3}{\frac{M!}{(M-\mathcal{W})!}} = \frac{\frac{(M-2)!}{(M-W)!}}{\frac{M!}{(M-\mathcal{W})!}} = \frac{1}{(M-1)M} \tag{2.38}$$

We summarize the distribution f in the following table:

$f(W_i, W_j)$	0	1	2	...	$\mathcal{W}$	$f_{W_j}(w_j)$
0	P_1	P_2	P_2	...	P_2	$P_1 + \mathcal{W}P_2$
1	P_2	0	P_3	...	P_3	$P_2 + (\mathcal{W}-1)P_3$
2	P_2	P_3	0	...	P_3	$P_2 + (\mathcal{W}-1)P_3$
$\vdots$	$\vdots$	$\vdots$	$\vdots$	...	$\vdots$	$\vdots$
$\mathcal{W}$	P_2	P_3	P_3	...	0	$P_2 + (\mathcal{W}-1)P_3$
$f_{W_i}(w_i)$	$P_1 + \mathcal{W}P_2$			...	$P_2 + (\mathcal{W}-1)P_3$	1

(2.39)

Calculating the covariance defined in the equation (1.42) by rows c_i, from the bivariate joint probability table, we have that $\text{Cov}(W_i, W_j) = c_0 + c_1 + \cdots + c_{\mathcal{W}}$ where

$$
\begin{aligned}
c_0 &= P_1(0-\mu_W)(0-\mu_W) + P_2(0-\mu_W)(1-\mu_W) + \cdots + P_2(0-\mu_W)(\mathcal{W}-\mu_W) \\
c_1 &= P_2(1-\mu_W)(0-\mu_W) + P_3(1-\mu_W)(2-\mu_W) + \cdots + P_3(1-\mu_W)(\mathcal{W}-\mu_W) \\
c_{\mathcal{W}} &= P_2(\mathcal{W}-\mu_W)(0-\mu_W) + P_3(\mathcal{W}-\mu_W)(1-\mu_W) + \cdots + P_3(\mathcal{W}-\mu_W)(\mathcal{W}-1-\mu_W)
\end{aligned}
$$

Resolving the sum and simplifying, we determine that the covariance of the scores random variable for Ranked-NoM coding is given by:

$$\text{Cov}(W_i, W_j) = \frac{\mu_W}{M-1}\left(\mu_W - \frac{2\mathcal{W}+1}{3}\right) \tag{2.40}$$

2.5 Modulation vector function

For (1.2), we denote $\Psi_H : \Omega \to \Xi_H$, the function for generating modulations vector for Ranked-NoM coding. The function we chose in the present article is parameterized by the number $\mathcal{N} \in \{1, \ldots, M\}$ and defined as:

$$\Psi_H(\hat{\mathbf{w}}^k; \mathcal{N}) \equiv \Phi_H(\hat{\mathbf{w}}^k; \mathcal{N}) \tag{2.41}$$

Below, we consider $\mathbf{v}_H^1 = \Psi_H(\hat{\mathbf{w}}^1; \mathcal{N}) = (\mathcal{N}\ (\mathcal{N}-1) \ldots 1\ 0 \ldots 0)^\mathsf{T}$.

For Ranked-NoM coding, the modulation vector gated up to the first $I < \mathcal{N}$ components is then defined by

$$\mathbf{v}_{H,I}^1 = (\mathcal{N}\ (\mathcal{N}-1) \ldots (\mathcal{N}-(I-1))\ 0 \ldots 0)^\mathsf{T} \tag{2.42}$$

Then, for $I = \mathcal{N}$, that is, for the final potential, the modulation vector is given by

$$\mathbf{v}_{H,\mathcal{N}}^1 = (\mathcal{N}\ (\mathcal{N}-1)\ \ldots\ 1\ 0\ \ldots\ 0)^\mathsf{T} \tag{2.43}$$

For $I > \mathcal{N}$, we also take $\mathbf{v}_{H,\mathcal{N}}^1$.

2.6 $S_{H,\mathcal{N}}$. Integration-Final Potential $I = N$

2.6.1 $\max(S_{H,\mathcal{N}})$

The maximum value of integration $S_{H,\mathcal{N}}$ is defined in equation (1.31). Thus for Ranked-NoM coding we have that $\mathbf{w}^1 = (\mathcal{W}, \mathcal{W}-1, \ldots, 1, 0, \ldots, 0)$ and modulation vector is given in the equation (2.43),

therefore $\max(S_{H,\mathcal{N}})$ is,

$$
\begin{aligned}
&=< \mathbf{w}^1, \mathbf{v}^1_{H,\mathcal{N}} > \\
&= \mathcal{W}\mathcal{N} + (\mathcal{W}-1)(\mathcal{N}-1) + \ldots + (\mathcal{W}-(\mathcal{N}-1))(\mathcal{N}-(\mathcal{N}-1)) \\
&= \mathcal{N}^2\mathcal{W} - (\mathcal{W}+\mathcal{N})\left(\frac{\mathcal{N}(\mathcal{N}+1)}{2} - \mathcal{N}\right) + \left(\frac{\mathcal{N}(\mathcal{N}+1)(2\mathcal{N}+1)}{6} - \mathcal{N}^2\right) \qquad (2.44)
\end{aligned}
$$

simplifying, the expression of the max value of integration is given by,

$$\max(S_{H,\mathcal{N}}) = \mathcal{W}\mathcal{N}\left(\frac{\mathcal{N}+1}{2}\right) + \frac{\mathcal{N}(1-\mathcal{N}^2)}{6} \qquad (2.45)$$

2.6.2 $\mathbf{E}[S_{H,\mathcal{N}}]$, expectation of final potential

$$
\begin{aligned}
\mathrm{E}[S_{H,\mathcal{N}}] &= \mathrm{E}[\mathbf{v}^{\mathsf{T}}_{H,\mathcal{N}}\,\mathbf{W}] = \mathbf{v}^{\mathsf{T}}_{H,\mathcal{N}}\,\mathrm{E}[\mathbf{W}] && (2.46)\\
&= \mathbf{v}^{\mathsf{T}}_{H,\mathcal{N}} \cdot \frac{1}{M}\left(\frac{\mathcal{W}(\mathcal{W}+1)}{2}, \ldots, \frac{\mathcal{W}(\mathcal{W}+1)}{2}\right)^{\mathsf{T}} && (2.47)\\
&= (\mathcal{N}\ (\mathcal{N}-1)\ \ldots\ 1\ 0\ \ldots\ 0) \cdot \frac{1}{M}\left(\frac{\mathcal{W}(\mathcal{W}+1)}{2}, \ldots, \frac{\mathcal{W}(\mathcal{W}+1)}{2}\right)^{\mathsf{T}} && (2.48)\\
&= \frac{1}{M}\left(\frac{\mathcal{N}\mathcal{W}(\mathcal{W}+1)}{2} + \frac{\mathcal{W}(\mathcal{N}-1)(\mathcal{W}+1)}{2} + \ldots + \frac{\mathcal{W}(\mathcal{W}+1)}{2}\right) \\
&= \frac{1}{M}\frac{\mathcal{W}(\mathcal{W}+1)}{2}(\mathcal{N} + (\mathcal{N}-1) + \ldots + 1) \\
&= \frac{1}{M}\frac{\mathcal{W}(\mathcal{W}+1)}{2}\frac{\mathcal{N}(\mathcal{N}+1)}{2} && (2.49)\\
&= \frac{1}{4M}\,\mathcal{W}(\mathcal{W}+1)\,\mathcal{N}(\mathcal{N}+1) && (2.50)
\end{aligned}
$$

Then the expectation of Integration $\mathrm{E}[S_{H,\mathcal{N}}]$ is given by

$$\mathrm{E}[S_{H,\mathcal{N}}] = \frac{1}{4M}\,\mathcal{W}(\mathcal{W}+1)\,\mathcal{N}(\mathcal{N}+1) \qquad (2.51)$$

2.6.3 $\mathbf{Var}[S_{H,\mathcal{N}}]$, variance of final potential

From (1.38), the variance of integration is given by $\mathrm{Var}[S_{H,\mathcal{N}}] = \mathbf{v}^{\mathsf{T}}_{H,\mathcal{N}} \cdot \mathbf{K}^{H}_{WW} \cdot \mathbf{v}_{H,\mathcal{N}}$.

Using (1.43) for the expression of Variance-covariance matrix $\mathbf{K}^H_{WW}$ and the modulation vector $\mathbf{v}^1_{H,\mathcal{N}}$ for final potential given by (2.43), we have :

$$\mathrm{Var}[S_{H,\mathcal{N}}] = \mathbf{v}^{\mathsf{T}}_{H,\mathcal{N}} \cdot \mathbf{K}^H_{WW} \cdot \mathbf{v}_{H,\mathcal{N}} \tag{2.52}$$

$$= (\mathcal{N}\ (\mathcal{N}-1)\ \ldots\ 1\ 0\ \ldots\ 0) \begin{bmatrix} \mathrm{Var}W & \vartheta & \ldots & \vartheta \\ \vartheta & \mathrm{Var}W & \ldots & \vartheta \\ \vdots & \vdots & \ddots & \vdots \\ \vartheta & \vartheta & \ldots & \mathrm{Var}W \end{bmatrix} \begin{bmatrix} \mathcal{N} \\ \mathcal{N}-1 \\ \vdots \\ 1 \\ 0 \\ \vdots \\ 0 \end{bmatrix} \tag{2.53}$$

$$= \mathcal{N}^2 \mathrm{Var}W + \mathcal{N}\ \vartheta \left(\frac{\mathcal{N}(\mathcal{N}+1)}{2} - \mathcal{N} \right) + (\mathcal{N}-1)^2 \mathrm{Var}W + \tag{2.54}$$

$$(\mathcal{N}-1)\ \vartheta \left(\frac{\mathcal{N}(\mathcal{N}+1)}{2} - (\mathcal{N}-1) \right) + \cdots + \vartheta \left(\frac{\mathcal{N}(\mathcal{N}+1)}{2} - 1) \right) + \mathrm{Var}W$$

$$= \mathrm{Var}W\ (\mathcal{N}^2 + (\mathcal{N}-1)^2 + \cdots + 1) + \vartheta \left[\mathcal{N} \left(\frac{\mathcal{N}(\mathcal{N}+1)}{2} - N \right) \right] + \tag{2.55}$$

$$\vartheta \left[(\mathcal{N}-1) \left(\frac{\mathcal{N}(\mathcal{N}+1)}{2} - (\mathcal{N}-1) \right) + \cdots + \left(\frac{\mathcal{N}(\mathcal{N}+1)}{2} - 1 \right) \right]$$

$$= \mathrm{Var}W \left(\frac{\mathcal{N}(\mathcal{N}+1)(2\mathcal{N}+1)}{6} \right) + \vartheta \left[\sum_{i=1}^{\mathcal{N}} i \left(\frac{\mathcal{N}(\mathcal{N}+1)}{2} - i \right) \right] \tag{2.56}$$

$$= \mathrm{Var}W\ \frac{\mathcal{N}(\mathcal{N}+1)}{2} \frac{2\mathcal{N}+1}{3} + \vartheta \left[\left(\frac{\mathcal{N}(\mathcal{N}+1)}{2} \right)^2 - \frac{\mathcal{N}(\mathcal{N}+1)}{2} \frac{2\mathcal{N}+1}{3} \right] \tag{2.57}$$

Denoting $p = \dfrac{\mathcal{N}(\mathcal{N}+1)}{2}$ and $q = \dfrac{2\mathcal{N}+1}{3}$, it reads:

$$\mathrm{Var}[S_{H,\mathcal{N}}] = (p\ q)\mathrm{Var}W\ + (p^2 - p\ q)\ \mathrm{Cov}(W_i, W_j) \tag{2.58}$$

where $\mathrm{Var}W$ is given by (2.27) and $\mathrm{Cov}(W_i, W_j)$ by (2.40).

In case $\mathcal{N} = \mathcal{W}$, we have:

$$\mathrm{Var}[S_{H,\mathcal{N}}] = \frac{M^2\ \mu_W^2}{M-1} (q - \mu_W)^2 \tag{2.59}$$

where μ_W is given by (2.21).

2.7 $S_{H,I}$. Integration-intermediate states, $I < \mathcal{N}$

The modulation vector gated up to the first I components is defined by $\mathbf{v}^1_{H,I} = (\mathcal{N}\ (\mathcal{N}-1) \ldots (\mathcal{N} - (I-1))\ 0 \ldots 0)^{\mathsf{T}}$ in equation (2.42).

2.7.1 $\mathrm{E}[S_{H,I}]$, expectation at intermediate states

$$
\begin{aligned}
\mathrm{E}[S_{H,I}] =& \mathrm{E}[\mathbf{v}_{H,I}^{\mathsf{T}}\,\mathbf{W}] = \mathbf{v}_{H,I}^{\mathsf{T}}\,\mathrm{E}[\mathbf{W}] && (2.60)\\
=& \mathbf{v}_{H,I}^{\mathsf{T}} \cdot \frac{1}{M}\left(\frac{\mathcal{W}(\mathcal{W}+1)}{2} \dots \frac{\mathcal{W}(\mathcal{W}+1)}{2}\right)^{\mathsf{T}} && (2.61)\\
=& (\mathcal{N}\ (\mathcal{N}-1)\dots(N-(I-1))\ 0\dots 0)\cdot && (2.62)\\
& \frac{1}{M}\left(\frac{\mathcal{W}(\mathcal{W}+1)}{2} \dots \frac{\mathcal{W}(\mathcal{W}+1)}{2}\right)^{\mathsf{T}} \\
=& \frac{\mathcal{W}(\mathcal{W}+1)}{2M}(\mathcal{N}+\mathcal{N}-1+\dots+\mathcal{N}-(I-1)) && (2.63)\\
=& \frac{\mathcal{W}(\mathcal{W}+1)}{2M}\left(I\,\mathcal{N}-\frac{I(I-1)}{2}\right) && (2.64)
\end{aligned}
$$

The expectation of Integration at intermediate states $S_{H,I}$ is then given by:

$$
\mathrm{E}[S_{H,I}] = \frac{I\mathcal{W}(\mathcal{W}+1)}{4M}(2\,\mathcal{N}-I+1) \tag{2.65}
$$

2.7.2 $\mathrm{Var}[S_{H,I}]$, variance at intermediate states

From (1.38), we have $\mathrm{Var}[S_{H,I}] = \mathbf{v}_{H,I}^{\mathsf{T}} \cdot \mathbf{K}_{WW}^{H} \cdot \mathbf{v}_{H,I}$. The Variance-covariance matrix $\mathbf{K}_{WW}^{H}$ is given by (1.43) and modulation vector $\mathbf{v}_{H,I}^{1}$ by (2.42). Therefore we have:

$$
\begin{aligned}
\mathrm{Var}[S_{H,I}] &= \mathbf{v}_{H,I}^{\mathsf{T}} \cdot \mathbf{K}_{WW}^{H} \cdot \mathbf{v}_{H,I} \\
&= (\mathcal{N}\ (\mathcal{N}-1)\ldots(\mathcal{N}-(I-1))\ 0\ldots 0) \cdot \mathbf{K}_{WW}^{H} \cdot \begin{bmatrix} \mathcal{N} \\ (\mathcal{N}-1) \\ \vdots \\ (\mathcal{N}-(I-1)) \\ 0 \\ \vdots \\ 0 \end{bmatrix} \qquad (2.66)\\
&= \mathrm{Var}W[\mathcal{N}^2 + (\mathcal{N}-1)^2 + \ldots + ((\mathcal{N}-(I-1))^2)] + \vartheta\,\mathcal{N} \qquad (2.67)\\
&[\mathcal{N} + \mathcal{N} - 1 + \ldots + (\mathcal{N}-(I-1)] + \vartheta\,(\mathcal{N}-1) \\
&[\mathcal{N} + \mathcal{N} - 2 + \ldots + (\mathcal{N}-(I-1)] + \ldots + \vartheta\,(\mathcal{N}-(I-1)) \\
&[\mathcal{N} + \mathcal{N} - 1 + \ldots + (\mathcal{N}-(I-2))] \\
&= \mathrm{Var}W\left[\sum_{i=0}^{I-1}(\mathcal{N}-i)^2\right] + \vartheta\left[\sum_{i=0}^{I-1}(\mathcal{N}-i)(\mathcal{N}(I-1))\right] - \qquad (2.68)\\
&\vartheta\left[\sum_{i=0}^{I-1}(\mathcal{N}-i)\left(\frac{I(I-1)}{2} - i\right)\right] \\
&= \mathrm{Var}W\left[\mathcal{N}^2 I - 2\,\mathcal{N}\frac{I(I-1)}{2} + \frac{I(I-1)}{2}\frac{(2\,I-1)}{3}\right] + \vartheta \qquad (2.69)\\
&\left[\mathcal{N}\frac{I(I-1)}{2}(2\,\mathcal{N} - I + 1)\right] - \vartheta \\
&\left[\frac{I(I-1)}{2}\left(\mathcal{N}(I-1) - \frac{I(I-1)}{2} + \frac{(2\,I-1)}{3}\right)\right]
\end{aligned}
$$

Defining $\hat{p} = \frac{I(I-1)}{2}$ and $\hat{q} = \frac{2\,I-1}{3}$ and simplifying, we obtain the formula for the variance at intermediate states:

$$
\begin{aligned}
\mathrm{Var}[S_{H,I}] = [\mathcal{N}(\mathcal{N}I - 2\hat{p}) + \hat{p}\,\hat{q}]\ \mathrm{VarW}+ \\
\hat{p}\,[2\mathcal{N}(\mathcal{N} - I + 1) + \hat{p} - \hat{q}]\ \mathrm{Cov}(W_i, W_j) \qquad (2.70)
\end{aligned}
$$

2.8 Behavior of discriminability for final potential

We set $\mathcal{N} = \mathcal{W} = M/2$

Let us define $p = \dfrac{\mathcal{N}(\mathcal{N}+1)}{2}$ and $q = \dfrac{2\mathcal{N}+1}{3}$, and substituting $\mathcal{N} = \mathcal{W}$, in each expression we get,

$$\max(S_{H,\mathcal{N}}) = \mathcal{W}\mathcal{N}\left(\frac{\mathcal{N}+1}{2}\right) + \frac{\mathcal{N}(1-\mathcal{N}^2)}{6} \tag{2.71}$$

$$= \mathcal{N}\mathcal{N}\left(\frac{\mathcal{N}+1}{2}\right) + \frac{\mathcal{N}(1+\mathcal{N})}{2}\frac{(1-\mathcal{N})}{3} \tag{2.72}$$

$$= \mathcal{N}\, p + p\,\frac{1-\mathcal{N}}{3} \tag{2.73}$$

$$= p\,(\mathcal{N} + \frac{1-\mathcal{N}}{3}) \tag{2.74}$$

$$= p\left(\frac{2\mathcal{N}+1}{3}\right) \tag{2.75}$$

$$= p\,q \tag{2.76}$$

$$\mathrm{E}[S_{H,\mathcal{N}}] = \lambda_C\,\mu_{W_C} \tag{2.77}$$

$$= \frac{\mathcal{N}(\mathcal{N}+1)}{2}\cdot\frac{\mathcal{W}(\mathcal{W}+1)}{2M} \tag{2.78}$$

$$= \frac{\mathcal{N}(\mathcal{N}+1)}{2}\cdot\frac{\mathcal{N}(\mathcal{N}+1)}{2M} \tag{2.79}$$

$$= \frac{p^2}{M} \tag{2.80}$$

For the variance $\mathrm{Var}[S_{H,\mathcal{N}}]$ we have that

$$\mathrm{Var} W_H = \mu_{W_H}\left(\frac{2\mathcal{W}+1}{3} - \mu_{W_H}\right) = \mu(q-\mu)$$

$$\mathrm{Cov}_H(W_i, W_j) = \frac{\mu_{W_H}}{M-1}\left(\mu_{W_H} - \frac{2\mathcal{W}+1}{3}\right) = \frac{\mu}{M-1}(\mu - q)$$

therefore,

$$\mathrm{Var}[S_{H,\mathcal{N}}] = (p\,q)\mathrm{Var}W \; + (p^2 - p\,q)\,\mathrm{Cov}(W_i, W_j) \tag{2.81}$$

$$= p\,q\,\mu(q-\mu) + (p^2 - p\,q)\,\frac{\mu}{M-1}(\mu - q) \tag{2.82}$$

$$= \frac{p\,\mu(q-\mu)(M\,q - p)}{M-1} \tag{2.83}$$

then substituting in the formula of discriminability, max (2.76), expectation(2.80) and variance (2.83) of integration for Ranked-NoM code, we get,

$$D_H(M) = \frac{\max(S_{H,\mathcal{N}}) - \mathrm{E}[S_{C,\mathcal{N}}]}{\sqrt{\mathrm{Var}[S_{C,\mathcal{N}}]}} \tag{2.84}$$

$$= \frac{p\,q - \dfrac{p^2}{M}}{\sqrt{\dfrac{p\,\mu(q-\mu)(M\,q-p)}{M-1}}} \tag{2.85}$$

$$= \sqrt{\frac{(p\,q - p^2/M)^2}{\dfrac{p\,\mu(q-\mu)(M\,q-p)}{M-1}}} \tag{2.86}$$

$$= \sqrt{\frac{p^2/M^2(qM-p)^2}{\dfrac{p\,\mu(q-\mu)(M\,q-p)}{M-1}}} \tag{2.87}$$

$$= \sqrt{\frac{(qM-p)(M-1)\,p}{M^2\mu(q-\mu)}} \tag{2.88}$$

we can see that we finally get:

$$D_H(M) = \sqrt{M-1} \tag{2.89}$$

3 APPLICATION TO N-OF-M (NOM) CODING

Since this scheme is already known as N-of-M coding, we keep the name. In our formalism, N will however become $\mathcal{W}$.

3.1 Scores vector function

The support Ω_F is generated by using the function of Ranked-NoM coding Φ_H. For NoM, Φ_F is a composition of the indicator function $\mathbf{1}_A$ with the Ranked-NoM function Φ_H where $A = \{w_i^k \in \Omega_H : w_i^k = \max(0, \hat{w}_i^k - M + \mathcal{W}) \neq 0\}$.

The scores are obtained by the vector-value function $\Phi_F(\hat{\mathbf{w}}^k) = \mathbf{w}_F^l$ defined in (1.1). Then the vectorial components are given by:

$$\phi_{F,i}(\hat{\mathbf{w}}^k) = \phi_{F,i}(\hat{w}_1^k, \dots, \hat{w}_M^k) = \mathbf{1}_A \circ \phi_{H,i}(\hat{w}_1^k, \dots, \hat{w}_M^k), \tag{3.1}$$

such as

$$\begin{aligned}
\Phi_F(\hat{\mathbf{w}}^k) &= \Phi_F(\hat{w}_1^k, \dots, \hat{w}_M^k) && (3.2)\\
&= (\phi_{F,1}(\hat{w}_1^k, \dots, \hat{w}_M^k), \dots, \phi_{F,M}(\hat{w}_1^k, \dots, \hat{w}_M^k)) && (3.3)\\
&= (\mathbf{1}_A(\phi_{H,1}(\hat{w}_1^k, \dots, \hat{w}_M^k)), \dots, \mathbf{1}_A(\phi_{H,M}(\hat{w}_1^k, \dots, \hat{w}_M^k))) && (3.4)\\
&= (\mathbf{1}_A(\max(0, \hat{w}_i^k - M + \mathcal{W}), \dots, (\mathbf{1}_A(\max(0, \hat{w}_i^k - M + \mathcal{W})))) && (3.5)\\
&= (w_1^l, \dots, w_M^l) = \mathbf{w}_F^l && (3.6)
\end{aligned}$$

Note that for $k = 1$, we have that $\hat{\mathbf{w}}^1 = (M, M-1\dots, 1)$, thus

$$\begin{aligned}
\Phi_F(\hat{\mathbf{w}}^1) &= (\mathbf{1}_A(\phi_{H,1}(\hat{w}_1^1, \dots, \hat{w}_M^1)), \dots, \mathbf{1}_A(\phi_{H,M}(\hat{w}_1^1, \dots, \hat{w}_M^1))) && (3.7)\\
&= (\mathbf{1}_A(\max(0, \mathcal{W})), \dots, \mathbf{1}_A(\max(0, \mathcal{W} - M + 1))) && (3.8)\\
&= (\mathbf{1}_A(\mathcal{W}), \mathbf{1}_A(\mathcal{W} - 1), \dots, \mathbf{1}_A(0)) && (3.9)\\
&= (1, 1, \dots, 0) = \mathbf{w}_F^1 && (3.10)
\end{aligned}$$

The vectors in the support of Ranked-NoM coding by the function Φ_F get the vectors in Ω_H converted into vectors of ones and zeros. Therefore, we have that Ω_F gets reduced vectors each time we generated vectors from Ω_H, because we are no longer interested in their order. Then we divide by the number of ways that you can arrange $\mathcal{W}$ numbers, which is $\mathcal{W}!$. Thus the cardinality of support of NoM, Ω_F is:

$$\frac{|\Omega_H|}{\mathcal{W}!} = \frac{M!}{\mathcal{W}!(M - \mathcal{W})!} = \binom{M}{\mathcal{W}} = |\Omega_F| \tag{3.11}$$

and thus $\Omega_F = \{\mathbf{w}^1, ..., \mathbf{w}^{|\Omega_F|}\}$.

Example 3.1. As an illustration, let $M = 4$ and $\mathcal{W} = 2$ ($|\Omega| = 24$, $|\Omega_H| = 12$ permutations and $|\Omega_F| = \binom{4}{2} = 6$ combinations.). We would have, for the best order:

$$\begin{aligned}
\Phi_F(\hat{\mathbf{w}}^1) &= (\mathbf{1}_A(\max(0, 2)), \mathbf{1}_A(\max(0, 1)), \mathbf{1}_A(\max(0, 0)), \mathbf{1}_A(\max(0, -1))) && (3.12)\\
&= (\mathbf{1}_A(2), \mathbf{1}_A(1), \mathbf{1}_A(0), \mathbf{1}_A(0))\\
&= (1, 1, 0, 0) = \mathbf{w}_H^1 && (3.13)
\end{aligned}$$

and for the worst order:

$$\begin{aligned}
\Phi_F(\hat{\mathbf{w}}^{24}) &= (\mathbf{1}_A(\max(0, -1)), \mathbf{1}_A(\max(0, 0)), \mathbf{1}_A(\max(0, 1)), \mathbf{1}_A(\max(0, 2))) && (3.14)\\
&= (\mathbf{1}_A(0), \mathbf{1}_A(0), \mathbf{1}_A(1), \mathbf{1}_A(2))\\
&= (0, 0, 1, 1) = \mathbf{w}_H^6 && (3.15)
\end{aligned}$$

Note that for $\hat{\mathbf{w}}^{18} = (2, 1, 3, 4)$ and $\hat{\mathbf{w}}^{23} = (1, 2, 4, 3)$ we would also obtain:

$$\begin{aligned}
\Phi_F(\hat{\mathbf{w}}^{18}) &= (\mathbf{1}_A(\max(0,0)), \mathbf{1}_A(\max(0,-1)), \mathbf{1}_A(\max(0,1)), \mathbf{1}_A(\max(0,2))) && (3.16)\\
&= (\mathbf{1}_A(0), \mathbf{1}_A(0), \mathbf{1}_A(1), \mathbf{1}_A(2))\\
&= (0,0,1,1) = \mathbf{w}_H^6 && (3.17)\\
\Phi_F(\hat{\mathbf{w}}^{23}) &= (\mathbf{1}_A(\max(0,-1)), \mathbf{1}_A(\max(0,0)), \mathbf{1}_A(\max(0,2)), \mathbf{1}_A(\max(0,1))) && (3.18)\\
&= (\mathbf{1}_A(0), \mathbf{1}_A(0), \mathbf{1}_A(2), \mathbf{1}_A(1))\\
&= (0,0,1,1) = \mathbf{w}_H^6 && (3.19)
\end{aligned}$$

therefore, $\Phi_F(\hat{\mathbf{w}}^{24}) = \Phi_F(\hat{\mathbf{w}}^{18}) = \Phi_F(\hat{\mathbf{w}}^{23}) = \mathbf{w}_H^6$.

3.2 Probability distribution of Scores

Let $\mathbf{W} = (W_1 \ W_2 \ \dots \ W_M)^\mathsf{T}$ be a discrete random vector with support the ordered set $\Omega_F = \{\mathbf{w}^1, \dots, \mathbf{w}^{\binom{M}{\mathcal{W}}}\}$, which is generated by the vector-function Φ_F defined in (3.1), thus the support is given by:

$$\Omega_F = \Omega_{W_1 W_2 \dots W_M} = \left\{ \begin{bmatrix} 1 \\ 1 \\ \vdots \\ 0 \end{bmatrix} \dots \begin{bmatrix} 0 \\ \vdots \\ 1 \\ 1 \end{bmatrix} \right\} \tag{3.20}$$

Due to (3.11), let its *joint probability mass function* be

$$P_{W_1 W_2 \dots W_M} = \begin{cases} \dfrac{1}{\binom{M}{\mathcal{W}}} = \dfrac{(M-\mathcal{W})!W!}{\mathcal{W}!} & \mathbf{w}^k \in \Omega_{W_1 W_2 \dots W_M} \\ 0 & \text{Otherwise} \end{cases} \tag{3.21}$$

From (1.17), the *marginal probability mass function* of $W_1, W_2, \dots, W_M$ with sample space $\mathcal{D}_{W_i} = \{1, 0\}$ for $i = 1, ..., M$ is given by:

$$P_{W_i}(w) = \begin{cases} \dfrac{\binom{M-1}{\mathcal{W}}}{\binom{M}{\mathcal{W}}} = \dfrac{M-\mathcal{W}}{M} & w = 0 \\ \dfrac{\binom{M-1}{\mathcal{W}-1}}{\binom{M}{\mathcal{W}}} = \dfrac{\binom{M-1}{\mathcal{W}-1}}{\frac{M}{\mathcal{W}}\binom{M-1}{\mathcal{W}-1}} = \dfrac{\mathcal{W}}{M} & w = 1 \\ 0 & \text{Otherwise} \end{cases} \tag{3.22}$$

3.3 Expectation vector of scores

3.3.1 $\boldsymbol{\mu_W}$, expected value of $\boldsymbol{W_i}$

$$\mathrm{E}[W] = \sum_{i=1}^{2} w_i \, P(w_i) = 1 \, \frac{\mathcal{W}}{M} = \frac{\mathcal{W}}{M} = \mu_W \tag{3.23}$$

3.3.2 $\mathbf{E[W]}$, expectation of $\mathbf{W}$,

Therefore, the expected value of random vector $\mathbf{W}$ is:

$$\mathrm{E}[\mathbf{W}] = \begin{bmatrix} E[W_1] \\ E[W_2] \\ \vdots \\ E[W_M] \end{bmatrix} = \begin{bmatrix} \frac{\mathcal{W}}{M} \\ \vdots \\ \frac{\mathcal{W}}{M} \end{bmatrix} \tag{3.24}$$

3.4 Variance-Covariance matrix of scores

The matrix $\mathbf{K}^F_{WW}$ is defined in (1.43) then we will find the variance Var$W$ and the covariance $\mathrm{Cov}(W_i, W_j) = \vartheta$.

3.4.1 Var$\boldsymbol{W}$, variance of $\boldsymbol{W_i}$

$$\mathrm{Var}(W) = \mathrm{E}[W^2] - \mathrm{E}[W]^2 \tag{3.25}$$

$$= \sum w_i^2 P(w_i) - \left(\frac{\mathcal{W}}{M}\right)^2 \tag{3.26}$$

$$= \frac{\mathcal{W}}{M} - \left(\frac{\mathcal{W}}{M}\right)^2 = \mu_W(1 - \mu_W) \tag{3.27}$$

Thus the variance of the random variable W is given by,

$$\mathrm{Var}(W) = \mu_W(1 - \mu_W) \tag{3.28}$$

3.4.2 $\boldsymbol{\vartheta}$, covariance of $\boldsymbol{W_i, W_j}$

From equations(3.21) and (3.22), the score random variable W_i of NoM coding are not independent because:

$$\prod_{i=1}^{M} P_{W_i}(w) = \left(\frac{M - \mathcal{W}}{M}\right)^{M-\mathcal{W}} \left(\frac{\mathcal{W}}{M}\right)^{\mathcal{W}} \neq \frac{(M - \mathcal{W})!\mathcal{W}!}{M!} = P_{W_1 W_2 \ldots W_M} \tag{3.29}$$

To find the covariance (1.42), we must find the bivariate joint probability distribution $f(W_i = w_i, W_j = w_j)$, for $i, j = 1, 2, \ldots, M$.

We have the following facts:

- Each random variable can take on 2 same binary values: $0, 1$.
- For any combination of w_i, w_j with $i, j = 1, \ldots M$ the joint probability distribution f are the same.
- The support Ω_F which is the sample space consists of $|\Omega_F| = \binom{M}{\mathcal{W}}$ random possible pair outcomes that are equally likely, that is, the probability for each random outcome is $\frac{1}{|\Omega_F|}$.
- $f(W_i, W_j) = f(W_j, W_i)$

To establish the joint probability distribution f, we need a general method to find the frequency of the various pair outcomes. As we have the fact that the random pair outcomes are equally likely, we only need to count the number of times each pair repeats.

Let us introduce a notation to define the support Ω_F (equation (3.20)) as a matrix and from here we get the frequency hence the joint probability distribution.

Definition 3.1. Let $\widehat{\binom{M}{\mathcal{W}}}$ being a $M \times \binom{M}{\mathcal{W}}$ matrix. $\binom{M}{k}_1$ denote the number of 1's and $\binom{M}{k}_0$ the number of 0's at the matrix $\widehat{\binom{M}{\mathcal{W}}}$. $[\binom{\mathcal{M}}{k}]$ denote a $\mathcal{M} \times \binom{\mathcal{M}}{k}$ matrix that represents all the combinations $\mathcal{M}$ choose k. For $k = 0$, $[\binom{\mathcal{M}}{0}]$ is a $\mathcal{M} \times 1$ column of zeros, and $k = \mathcal{M}$, $[\binom{\mathcal{M}}{\mathcal{M}}]$ is a $\mathcal{M} \times 1$ column of ones.

To build the support Ω_F as a matrix, we first set the number one in the first row of the matrix and then set the zero. We know that the sum of ones and zeros is $|\Omega_F| = \binom{M}{\mathcal{W}}$. To know how many numbers one $(1's)$ we have, we use the combination formula for one less "1" and one less "element" of the vector. Using the notation above we will have $\binom{M-1}{\mathcal{W}-1}_1$ ones $(1's)$.

The number of zeros $(0's)$ is $\binom{M-1}{\mathcal{W}}_0$ since the size of the vector decreases but the ones $(1's)$ that are W, remain fixed. So we have $\binom{M-1}{\mathcal{W}-1}_1 + \binom{M-1}{\mathcal{W}}_0 = \binom{M}{\mathcal{W}}$. In the second row we set the ones and zeros again in the same way but for each row of ones and zeros, and so on.

In general, the matrix $\widehat{\binom{M}{\mathcal{W}}}$ can be represented as a tree, where each line j represents the jth row of the matrix

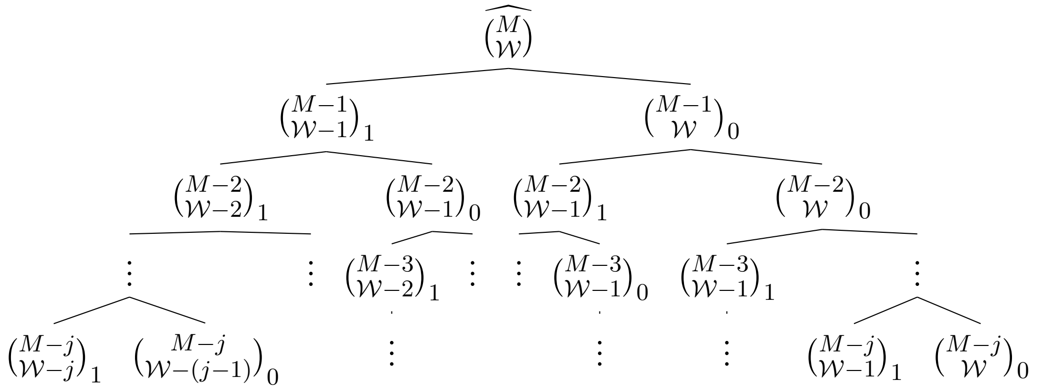

Tree for NoM coding (3.30)

Note that the last row using the tree is when $\mathcal{W} = j$ but it is not the last row of the matrix. We define the matrices $[\binom{\mathcal{M}}{k}]$, to denote the entire matrix in the definition (3.1).

For instance, with $M = 4, \mathcal{W} = 2$ we have

$$\Omega_{W_1W_2W_3W_4} = \widehat{\binom{4}{2}} = \begin{bmatrix} & \binom{3}{1}_1 & & & \binom{3}{2}_0 & \\ \binom{2}{0}_1 & & \binom{2}{1}_0 & \binom{2}{1}_1 & & \binom{2}{2}_0 \\ [\binom{2}{0}] & & [\binom{2}{1}] & [\binom{2}{1}] & & [\binom{2}{2}] \end{bmatrix} \tag{3.31}$$

$$= \begin{bmatrix} 1 & 1 & 1 & 0 & 0 & 0 \\ 1 & 0 & 0 & 1 & 1 & 0 \\ 0 & 1 & 0 & 1 & 0 & 1 \\ 0 & 0 & 1 & 0 & 1 & 1 \end{bmatrix}_{4\times\binom{4}{2}} \tag{3.32}$$

the first row corresponds to the first random variable W_1, the second row to the W_2 random variable and so on.

To find the frequency we can consider two first random variables W_1, W_2 in Ω_F,

$$\Omega_{W_1 \ldots W_M} = \widehat{\binom{M}{\mathcal{W}}} = \begin{bmatrix} & \binom{M-1}{\mathcal{W}-1}_1 & & & \binom{M-1}{\mathcal{W}}_0 & \\ \binom{M-2}{\mathcal{W}-2}_1 & & \binom{M-2}{\mathcal{W}-1}_0 & \binom{M-2}{\mathcal{W}-1}_1 & & \binom{M-2}{\mathcal{W}}_0 \\ \vdots & & \vdots & \vdots & & \vdots \end{bmatrix} \tag{3.33}$$

$$= \begin{bmatrix} & \binom{M-1}{\mathcal{W}-1}_1 & & & \binom{M-1}{\mathcal{W}}_0 & \\ f_3 & & f_2 & f_2 & & f_1 \\ \vdots & & \vdots & \vdots & & \vdots \end{bmatrix} \tag{3.34}$$

the frequency of the pair outcome $(1,1)$ is $\binom{M-2}{\mathcal{W}-2}_1 = f_3$, of the pair $(1,0) = (0,1)$ is $\binom{M-2}{\mathcal{W}-1}_0 = \binom{M-2}{\mathcal{W}-1}_1 = f_2$ and $(0,0)$ is $\binom{M-2}{\mathcal{W}}_0 = f_1$ therefore we have that,

$$f(W_1, W_1) = P_1 = \frac{f_1}{\binom{M}{\mathcal{W}}} = \frac{\binom{M-2}{\mathcal{W}}}{\binom{M}{\mathcal{W}}} \tag{3.35}$$

$$f(W_1, W_2) = P_2 = \frac{f_2}{\binom{M}{\mathcal{W}}} = \frac{\binom{M-2}{\mathcal{W}-1}}{\binom{M}{\mathcal{W}}} \tag{3.36}$$

$$f(W_2, W_2) = P_3 = \frac{f_3}{\binom{M}{\mathcal{W}}} = \frac{\binom{M-2}{\mathcal{W}-2}}{\binom{M}{\mathcal{W}}} \tag{3.37}$$

We summarize the bivariate joint probability distribution f in the following table,

$f(W_i, W_j)$	0	1	$f_{W_j}(W_j)$
0	P_1	P_2	$P_1 + P_2$
1	P_2	P_3	$P_2 + P_3$
$f_{W_i}(W_i)$	$P_1 + P_2$	$P_2 + P_3$	1

(3.38)

Now we can calculate the covariance(1.42). Doing the operations by rows, we have that $\mathrm{Cov}(W_i, W_j) = c_0 + c_1$ where

$$c_0 = P_1\,(0 - \mu_W)(0 - \mu_W) + P_2\,(0 - \mu_W)(1 - \mu_W) \tag{3.39}$$

$$c_1 = P_2\,(1 - \mu_W)(0 - \mu_W) + P_3\,(1 - \mu_W)(1 - \mu_W) \tag{3.40}$$

Using the combination formula,

$$\binom{n}{r} = \frac{n!}{r!(n-r)!}$$

the following properties of the combination

$$\binom{n}{1} = n, \quad \binom{n}{0} = \binom{n}{n} = 1, \quad \binom{n}{n-r} = \binom{n}{r}, \quad \binom{n}{r} + \binom{n}{r-1} = \binom{n+1}{r} \tag{3.41}$$

and simplifying, we get,

$$\mathrm{Cov}(W_i, W_j) = c_0 + c_1 \tag{3.42}$$
$$= P_1\,\mu_W^2 + P_2\,\mu_W(1-\mu_W) - P_2\,\mu_W(1-\mu_W) + P_3\,(1-\mu_W)^2 \tag{3.43}$$
$$= \mu_W^2 - 2\mu_W\,P_3\frac{\mathcal{W}-1}{M-1} + P_3 \tag{3.44}$$
$$= \mu_W\frac{\mathcal{W}-1}{M-1} - \mu_W^2 \tag{3.45}$$

Thus the covariance $\mathrm{Cov}(W_i, W_j)$ of scores of NoM is given by:

$$\mathrm{Cov}(W_i, W_j) = \mu_W\frac{\mathcal{W}-1}{M-1} - \mu_W^2 \tag{3.46}$$

where μ_W is defined in the equation (3.23).

3.5 Modulation vector function

For (1.2), we denote $\Psi_F : \Omega \to \Xi_F$, the function for generating modulations vector for NoM coding. The function we chose in the present article is parameterized by the number $\mathcal{N} \in \{1,\ldots,M\}$ and defined as:

$$\Psi_F(\hat{\mathbf{w}}^k; \mathcal{N}) \equiv \Phi_F(\hat{\mathbf{w}}^k; \mathcal{N}) \tag{3.47}$$

Below, we consider $\mathbf{v}_F^1 = \Psi_F(\hat{\mathbf{w}}^1; \mathcal{N}) = (1,\ldots,1_{\mathcal{N}},0,\ldots 0)^\mathsf{T}$, which contains $\mathcal{N}$ ones.

For NoM, the modulation vector gated up to the first I components is then defined by

$$\mathbf{v}_{F,I}^1 = (1,\ldots,1,0,\ldots 0)^\mathsf{T} \tag{3.48}$$

which contains $I < \mathcal{N}$ ones.

3.6 $S_{F,\mathcal{N}}$, integration-Final Potential $I = N$

3.6.1 max($S_{F,\mathcal{N}}$)

The maximum value of integration $S_{F,\mathcal{N}}$ is defined in equation (1.31). Thus, for NoM coding, we have that $\mathbf{w}^1 = (1,\ldots,1_{\mathcal{W}},0,\ldots,0)$ and modulation vector is given in the equation (3.48), therefore,

$$\max(S_{F,\mathcal{N}}) = 1 + 1 \ldots + 1 = \mathcal{N} \tag{3.49}$$

considering $\mathcal{W} > \mathcal{N}$. If $\mathcal{W} < \mathcal{N}$, $\max(S_{F,\mathcal{N}}) = \mathcal{W}$.

3.6.2 $\mathrm{E}[S_{F,\mathcal{N}}]$, expectation of final potential

$$\mathrm{E}[S_{F,\mathcal{N}}] = \mathrm{E}[\mathbf{v}_{F,I}^\mathsf{T}\,\mathbf{W}] = \mathbf{v}_{F,I}^\mathsf{T}\,\mathrm{E}[\mathbf{W}] \tag{3.50}$$
$$= \mathbf{v}_{F,I}^\mathsf{T} \cdot \left(\frac{\mathcal{W}}{M},\ldots,\frac{\mathcal{W}}{M}\right)^\mathsf{T} \tag{3.51}$$
$$= (1\;1\;\ldots\;0) \cdot \left(\frac{\mathcal{W}}{M},\ldots,\frac{\mathcal{W}}{M}\right)^\mathsf{T}$$
$$= \left(\frac{\mathcal{W}}{M} + \ldots + \frac{\mathcal{W}}{M}\right) = \frac{\mathcal{N}\,\mathcal{W}}{M} \tag{3.52}$$

Then the expectation of Integration $S_{F,\mathcal{N}}$ is given by

$$\mathrm{E}[S_{F,\mathcal{N}}] = \frac{\mathcal{N}\,\mathcal{W}}{M} \tag{3.53}$$

3.6.3 $\mathbf{Var}[S_{F,\mathcal{N}}]$, variance of final potential

From (1.38), we have $\mathrm{Var}[S_{F,\mathcal{N}}] = \mathbf{v}_{F,\mathcal{N}}^{\mathsf{T}} \cdot \mathbf{K}_{WW}^{F} \cdot \mathbf{v}_{F,\mathcal{N}}$. The Variance-covariance matrix, $\mathbf{K}_{WW}^{F}$, is defined in equation (1.43), and the modulation vector $\mathbf{v}_{F,\mathcal{N}}^{1}$ of final potential for NoM coding is given by (3.48). Therefore,

$$\mathrm{Var}[S_{F,\mathcal{N}}] = \mathbf{v}_{F,\mathcal{N}}^{\mathsf{T}} \cdot \mathbf{K}_{WW}^{F} \cdot \mathbf{v}_{F,\mathcal{N}} \tag{3.54}$$

$$= (1\,1\ldots 1\,0\,\ldots 0)\begin{bmatrix} \mathrm{Var}W & \vartheta & \ldots & \vartheta \\ \vartheta & \mathrm{Var}W & \ldots & \vartheta \\ \vdots & \vdots & \ddots & \vdots \\ \vartheta & \vartheta & \ldots & \mathrm{Var}W \end{bmatrix}\begin{bmatrix}1\\1\\ \vdots \\ 1\\0\\ \vdots\\0\end{bmatrix} \tag{3.55}$$

$$= \mathrm{Var}W + (\mathcal{N}-1)\,\vartheta + \ldots + \mathrm{Var}W + (\mathcal{N}-1)\,\vartheta \tag{3.56}$$

$$= \mathcal{N}\;\mathrm{Var}W + \mathcal{N}\;(\mathcal{N}-1)\vartheta \tag{3.57}$$

Then the variance of integration is given by:

$$\mathrm{Var}[S_{F,\mathcal{N}}] = \mathcal{N}\;\mathrm{Var}W + \mathcal{N}\;(\mathcal{N}-1)\;\mathrm{Cov}(W_i, W_j) \tag{3.58}$$

where $\mathrm{Var}W$ is given by (3.28) and $\mathrm{Cov}(W_i, W_j)$ by (3.46).

We check that $\mathrm{Var}[S_{F,\mathcal{N}}]$ equation (3.58) is equivalent to the variance considering that NoM model follows the hypergeometric distribution:

$$\mathrm{Var}[S_{F,\mathcal{N}}] = \mathcal{N}\;\mathrm{Var}(W) + \mathcal{N}\;(\mathcal{N}-1)\;\mathrm{Cov}(W_i, W_j) \tag{3.59}$$

$$= \mathcal{N}(\mu_W(1-\mu_W)) + \mathcal{N}\;(\mathcal{N}-1)\left(\mu_W \frac{\mathcal{W}-1}{M-1} - \mu_W^2\right) \tag{3.60}$$

$$= N\;\mu_W[1-\mu_W + (\mathcal{N}-1)\frac{\mathcal{W}-1}{M-1} - (\mathcal{N}-1)\mu_W] \tag{3.61}$$

$$= \mathcal{N}\;\mu_W\left[1 - \frac{\mathcal{W}\,\mathcal{N}}{M} + \frac{(\mathcal{N}-1)(\mathcal{W}-1)}{M-1}\right] \tag{3.62}$$

$$= \frac{\mathcal{W}\,\mathcal{N}}{M}\left[\frac{(M-\mathcal{N})(M-\mathcal{W})}{M(M-1)}\right] \tag{3.63}$$

3.7 $S_{F,I}$. Integration-intermediate states, $I < \mathcal{N}$

3.7.1 $\mathrm{E}[S_{F,I}]$, expectation at intermediate states

$$\mathrm{E}[S_{F,I}] = \mathrm{E}[\mathbf{v}_{F,I}^{\mathsf{T}}\,\mathbf{W}] = \mathbf{v}_{F,I}^{\mathsf{T}}\,\mathrm{E}[\mathbf{W}] \tag{3.64}$$

$$= \mathbf{v}_{F,I}^{\mathsf{T}} \cdot \left(\frac{\mathcal{W}}{M}, \ldots, \frac{\mathcal{W}}{M}\right)^{\mathsf{T}}$$

$$= (1\ 1\ \ldots\ 0) \cdot \left(\frac{\mathcal{W}}{M}, \ldots, \frac{\mathcal{W}}{M}\right)^{\mathsf{T}}$$

$$= \left(\frac{\mathcal{W}}{M} + \ldots + \frac{\mathcal{W}}{M}\right) = \frac{I\,\mathcal{W}}{M} \tag{3.65}$$

Then the expectation of Integration $S_{F,I}$ is given by

$$\mathrm{E}[S_{F,I}] = \frac{I\,\mathcal{W}}{M} \tag{3.66}$$

3.7.2 $\mathbf{Var}[S_{F,I}]$, variance at intermediate states

From (1.38), the variance at intermediate states is given by $\mathrm{Var}[S_{F,I}] = \mathbf{v}_{F,I}^{\mathsf{T}} \cdot \mathbf{K}_{WW}^{F} \cdot \mathbf{v}_{F,I}$. From (1.43), we have the Variance-covariance matrix $\mathbf{K}_{WW}^{F}$ and the modulation vector $\mathbf{v}_{F,I}^{1}$ for NoM code is given by (3.48). Therefore,

$$\mathrm{Var}[S_{F,I}] = \mathbf{v}_{F,I}^{\mathsf{T}} \cdot \mathbf{K}_{WW}^{F} \cdot \mathbf{v}_{F,I}$$

$$= (1 \ldots 1\ 0\ \ldots 0) \begin{bmatrix} \mathrm{Var}W & \vartheta & \ldots & \vartheta \\ \vartheta & \mathrm{Var}W & \ldots & \vartheta \\ \vdots & \vdots & \ddots & \vdots \\ \vartheta & \vartheta & \ldots & \mathrm{Var}W \end{bmatrix} \begin{bmatrix} 1 \\ \vdots \\ 1 \\ 0 \\ \vdots \\ 0 \end{bmatrix} \tag{3.67}$$

$$= \mathrm{Var}W + (I-1)\,\vartheta + \ldots + \mathrm{Var}W + (I-1)\,\vartheta \tag{3.68}$$

$$= I\ \mathrm{Var}W + I\ (I-1)\vartheta \tag{3.69}$$

Then the variance of integration is given by

$$\mathrm{Var}[S_{F,I}] = I\ \mathrm{Var}W + I\ (I-1)\ \mathrm{Cov}(W_i, W_j) \tag{3.70}$$

where $\mathrm{Var}W$ is given by (3.28) and $\mathrm{Cov}(W_i, W_j)$ by (3.46).

3.8 Behavior of discriminability for final potential

Substituting $\mathcal{N} = \mathcal{W} = M/2$, we have,

$$\max(S_{F,\mathcal{N}}) = \mathcal{N} = \frac{M}{2} \tag{3.71}$$

$$\mathrm{E}[S_{F,\mathcal{N}}] = \lambda_F\ \mu_{W_F} \tag{3.72}$$

$$= \mathcal{N}\frac{\mathcal{W}}{M} = \frac{M}{4} \tag{3.73}$$

thus for $\mathcal{N} = M/2$ and simplifying we get, For the variance $\mathrm{Var}[S_{F,\mathcal{N}}]$, we have that

$$\mathrm{Var} W_F = \mu_{W_F}\left(1 - \mu_{W_F}\right) = \frac{\mathcal{W}}{M}\left(1 - \frac{\mathcal{W}}{M}\right) = 1/4$$

$$\mathrm{Cov}_H(W_i, W_j) = \frac{\mu_{W_H}}{M-1}\left(\mu_{W_H} - \frac{2\mathcal{W}+1}{3}\right) = \frac{1}{4(1-M)}$$

therefore,

$$\mathrm{Var}[S_{F,\mathcal{N}}] = \mathcal{N}\mathrm{Var}W \ + \mathcal{N}(\mathcal{N}-1)\,\mathrm{Cov}(W_i, W_j) \tag{3.74}$$

$$= \frac{M}{2}\frac{1}{4} + \frac{M}{4}(M-2)\,\frac{1}{4(1-M)} \tag{3.75}$$

$$= \frac{M^2}{16(M-1)} \tag{3.76}$$

then substituting in the formula of discriminability, max (3.71), expectation(3.73) and variance (3.76) of integration for NoM code, we get a function depending on M,

$$D_F(M) = \frac{\max(S_{F,\mathcal{N}}) - \mathrm{E}[S_{F,\mathcal{N}}]}{\sqrt{\mathrm{Var}[S_{F,\mathcal{N}}]}} \tag{3.77}$$

$$= \frac{\frac{M}{2} - \frac{M}{4}}{\sqrt{\frac{M^2}{16(M-1)}}} \tag{3.78}$$

$$= \sqrt{M-1} \tag{3.79}$$

therefore,

$$D_F(M) = \sqrt{M-1} \tag{3.80}$$

4 APPLICATION TO RANK-ORDER CODING

4.1 Scores vector function

Rank-Order Coding weights have no other parameter than M. Under this coding, we want the best weights vector $\mathbf{w^1} = \hat{\mathbf{w}}^\mathbf{1} = \Phi(\mathcal{R}(1))$ made up of an arithmetic sequence from M down to zero, with rate -1. The weights are obtained by the vector-value function $\Phi_R(\hat{\mathbf{w}}^k) = \mathbf{w}_R^k$ define in (1.1). We then define $\phi_{R,i}$ as the identity function,

$$\phi_{R,i}(\hat{\mathbf{w}}^k) = \hat{\mathbf{w}}^k = \mathbf{w}_R^k \tag{4.1}$$

such that:

$$\begin{aligned}\Phi_R(\hat{\mathbf{w}}^k) &= \Phi_R(M - r_1^k, M - r_2^k, \ldots, M - r_M^k) && (4.2)\\ &= (M - r_1^k, M - r_2^k, \ldots, M - r_M^k) && (4.3)\\ &= (w_1^k, \ldots, w_M^k) = \mathbf{w}_R^k && (4.4)\end{aligned}$$

Note that, for $k = 1$, we have that $\hat{\mathbf{w}}^1 = (M, M-1 \ldots, 1)$, given that $\mathbf{r}^1 = (r_1^1, \ldots, r_M^1) = (0, 1, \ldots, M-1)$. Thus

$$\begin{aligned}\Phi_R(\hat{\mathbf{w}}^1) &= \Phi_R(M, M-1, \ldots, 1) && (4.5)\\ &= (M, M-1, \ldots, 1) = (w_1^1, \ldots, w_M^1) = \mathbf{w}_R^1 && (4.6)\end{aligned}$$

For $k = M!$, given that $\mathbf{r}^{M!} = (r_1^{M!}, r_2^{M!}, \ldots, r_M^{M!}) = ((M-1), (M-2), \ldots, 0)$ we have that $\hat{\mathbf{w}}^{M!} = (1, 2, \ldots, M)$, so we get:

$$\begin{aligned}\Phi_R(\hat{\mathbf{w}}^{M!}) &= \Phi_R(1, 2, \ldots, M) && (4.7)\\ &= (1, 2, \ldots, M) = (w_1^{M!}, \ldots, w_M^{M!}) = \mathbf{w}_R^{M!} && (4.8)\end{aligned}$$

Φ_R being a bijection, we have:

$$|\Omega| = |\Omega_R| = M! \tag{4.9}$$

Example 4.1. As an illustration, let $M = 4$ ($|\Omega_C| = 24$ permutations). We would have, for the best order:

$$\Phi_R(\hat{\mathbf{w}}^1) = \Phi_R(4, 3, 2, 1) = (4, 3, 2, 1) = \mathbf{w}_R^1 \tag{4.10}$$

and for the worst order

$$\Phi_R(\hat{\mathbf{w}}^{24}) = \Phi_R(1, 2, 3, 4) = (1, 2, 3, 4) = \mathbf{w}_R^{24} \tag{4.11}$$

4.2 Probability distribution function of scores

Let $\mathbf{W} = (W_1\ W_2\ \ldots\ W_M)^\mathsf{T}$ be the discrete random vector with support the ordered set $\Omega_R = \{\mathbf{w}^1, \ldots, \mathbf{w}^{M!}\}$, which is generated by the vector-value function $\hat{\Phi}_R$ defined in (4.1), therefore,

$$\Omega_R = \Omega_{W_1 W_2 \ldots W_M} = \left\{ \begin{bmatrix} M \\ M-1 \\ \vdots \\ 1 \end{bmatrix} \begin{bmatrix} M-1 \\ M \\ \vdots \\ 1 \end{bmatrix} \cdots \begin{bmatrix} 1 \\ \vdots \\ M-1 \\ M \end{bmatrix} \right\} \tag{4.12}$$

Due to (4.9), its *joint probability mass function* is

$$P_{W_1 W_2 \dots W_M} = \begin{cases} \frac{1}{M!} & \mathbf{w}^k \in \Omega_{W_1 W_2 \dots W_M} \\ 0 & \text{Otherwise} \end{cases} \tag{4.13}$$

and from (1.17), the *marginal probability mass function* of $W_1, W_2, \dots, W_M$ with sample space $\mathcal{D}_{W_i} = \{M, M-1, ..., 1\}$ for $i = 1, ..., M$, is given by:

$$P_{W_i}(w) = \begin{cases} \frac{(M-1)!}{M!} = \frac{1}{M} & w = 1, \dots, M \\ 0 & \text{Otherwise} \end{cases} \tag{4.14}$$

4.3 Expectation vector of scores

4.3.1 $\boldsymbol{\mu_W}$, expected value of $\boldsymbol{W_i}$

$$\mathrm{E}[W] = \sum_{i=1}^{M} w_i \, P(w_i) = \sum_{i=1}^{M} w_i \, \frac{1}{M} \tag{4.15}$$

$$= \frac{1}{M} \sum_{i=1}^{M} w_i = \frac{1}{M} \frac{M(M+1)}{2} = \frac{(M+1)}{2} \tag{4.16}$$

4.3.2 $\mathbf{E[W]}$, expectation of $\mathbf{W}$

Therefore, the expected value of a random vector $\mathbf{W}$ is:

$$\mathrm{E}[\mathbf{W}] = \begin{bmatrix} E[W_1] \\ E[W_2] \\ \vdots \\ E[W_M] \end{bmatrix} = \begin{bmatrix} \frac{M+1}{2} \\ \vdots \\ \frac{M+1}{2} \end{bmatrix} \tag{4.17}$$

4.4 Variance-Covariance matrix of scores

The matrix $\mathbf{K}^R_{WW}$ is defined in (1.43) then we will find the variance Var$W$ and the covariance $\mathrm{Cov}(W_i, W_j) = \vartheta$.

4.4.1 Var$\boldsymbol{W}$, variance of $\boldsymbol{W_i}$

$$\mathrm{Var}(W) = \mathrm{E}[W^2] - \mathrm{E}[W]^2 \tag{4.18}$$

$$= \sum w_i^2 P(w_i) - \frac{(M+1)^2}{4} \tag{4.19}$$

$$= \sum w_i^2 \frac{1}{M} - \frac{(M+1)^2}{4}$$

$$= \frac{1}{M} \left(\frac{M(M+1)(2M+1)}{6} \right) - \frac{(M+1)^2}{4} \tag{4.20}$$

$$= \frac{M^2 - 1}{12} \tag{4.21}$$

thus the variance of random variable W is given by,

$$\mathrm{Var}W = \frac{M^2-1}{12} = \frac{\mu_W\,(M-1)}{6} \tag{4.22}$$

4.4.2 ϑ, covariance of $\boldsymbol{W_i}, \boldsymbol{W_j}$

From (4.13) and (4.14), the score random variable W_i of ROC coding are not independent because:

$$\prod_{i=1}^{M} P_{W_i}(w) = \left(\frac{1}{M}\right)^M \neq \frac{1}{M!} = P_{W_1 W_2 \ldots W_M} \tag{4.23}$$

hence $\mathrm{Cov}(W_i, W_j) \neq 0$.

From (1.42),we must find the bivariate joint probability distribution $f(W_i = w_i, W_j = w_j), i, j = 1, 2, \ldots, M$ to find the covariance. We will use the following facts:

- Each random variable can take on M same values: $1, 2, \ldots, M$
- For any combination of w_i, w_j with $i, j = 1, \ldots M$ the joint probability distribution f are the same.
- The support Ω_R which is the sample space consists of $|\Omega_R| = M!$ possible pair outcomes that are equally likely, that is, the probability for each outcome is $\frac{1}{|\Omega_R|}$.
- $f(W_i, W_j) = f(W_j, W_i)$
- $f(W_i = w_i, W_j = w_j) = 0$, for $i = j$

To obtain the support Ω_R (4.12) as a matrix where the rows would represent the random variables $W_1, W_2, \ldots, W_M$, we consider all permutations of the set $(M, M-1, \ldots, 1)$, and arrange them as columns in decreasing lexical order.

For example, for $M = 3$ the support represented as a matrix is given by:

$$\Omega_1 = \begin{bmatrix} 3 & 3 & 2 & 2 & 1 & 1 \\ 2 & 1 & 3 & 1 & 3 & 2 \\ 1 & 2 & 1 & 3 & 2 & 3 \end{bmatrix}_{3\times 3!} \tag{4.24}$$

and for $M = 4$ we have that,

$$\Omega_2 = \begin{bmatrix} 4 & 4 & 4 & 4 & 4 & 4 & \ldots \\ 3 & 3 & 2 & 2 & 1 & 1 & \ldots \\ 2 & 1 & 3 & 1 & 3 & 2 & \ldots \\ 1 & 2 & 1 & 3 & 2 & 3 & \ldots \end{bmatrix}_{4\times 4!} \tag{4.25}$$

Let us consider the two first variables W_1, W_2. If we fix the value for W_1, we are left with the permutations of the $M-1$ remaining values. Hence, each value appears $(M-1)!$ times. If we now fix also the value for W_2, we are left with the permutations of the $M-2$ remaining values. Hence, we have :

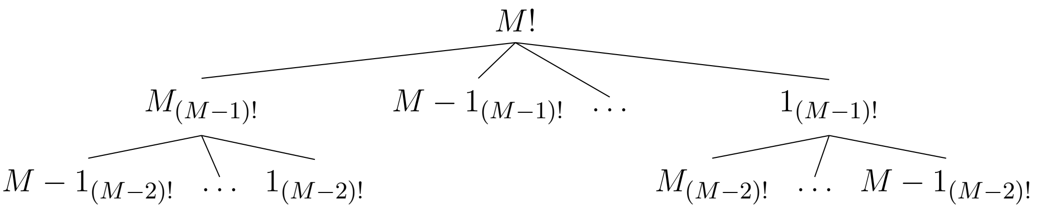

Tree for ROC coding (4.26)

Therefore, the frequency is the same for each possible pair outcome, and $f = (M-2)!$. Then the probability P is given by:

$$P = \frac{(M-2)!}{M!} = \frac{(M-2)!}{(M-2)!\,(M-1)\,M} = \frac{1}{(M-1)\,M} \tag{4.27}$$

We summarize the distribution f in the following table,

$f(W_i, W_j)$	1	2	$\ldots$	M	$f_{W_j}(W_j)$
1	0	P	$\ldots$	P	$(M-1)P$
2	P	0	$\ldots$	P	$(M-1)P$
$\vdots$	$\vdots$	$\vdots$	$\ldots$	$\vdots$	$\vdots$
M	P	P	$\ldots$	0	$(M-1)P$
$f_{W_i}(w_i)$	$(M-1)P$	$(M-1)P$	$\ldots$		1

(4.28)

To calculate the covariance defined in the equation (1.42), we do the operations by rows $c_1, \ldots, c_M$ from the bivariate joint distribution, and obtain:

$$\begin{aligned} c_1 &= (1-\mu_W)(2-\mu_W)\,P + (1-\mu_W)(3-\mu_W)\,P + \ldots + (1-\mu_W)(M-\mu_W)\,P \\ c_2 &= (2-\mu_W)(1-\mu_W)\,P + (2-\mu_W)(3-\mu_W)\,P + \ldots + (2-\mu_W)(M-\mu_W)\,P \\ &\ldots \\ c_M &= (M-\mu_W)(1-\mu_W)\,P + \ldots + (M-\mu_W)((M-1)-\mu_W)\,P \end{aligned}$$

Therefore $\text{Cov}(W_i, W_j) = c_1 + c_2 + \ldots + c_M$,

$$\begin{aligned} \text{Cov}(W_i, W_j) =& (1-\mu_W)\,P[(2-\mu_W) + \ldots + (M-\mu_W)] + \\ & (2-\mu_W)\,P[(1-\mu_W) + (3-\mu_W) \ldots + (M-\mu_W) + \\ & (M-\mu_W)\,P[(1-\mu_W) + (2-\mu_W) \ldots + ((M-1)-\mu_W)] \end{aligned} \tag{4.29}$$

$$\begin{aligned} =& (1-\mu_W)\,P\left[\frac{M(M+1)}{2} - 1 - (M-1)\mu_W\right] + \\ & (2-\mu_W)\,P\left[\frac{M(M+1)}{2} - 2 - (M-1)\mu_W\right] + \\ & (M-\mu_W)\,P\left[\frac{M(M+1)}{2} - M - (M-1)\mu_W\right] \end{aligned} \tag{4.30}$$

$$= \sum_{i=1}^{M} (i-\mu_W)\,P\,[M\,\mu_W - i - (M-1)\mu_W] \tag{4.31}$$

$$= \sum_{i=1}^{M} (i-\mu_W)\,P\,(\mu_W - i) = -P\,\sum_{i=1}^{M} (i-\mu_W)^2 \tag{4.32}$$

Using the summation formulas and simplifying, we finally get that the covariance of the scores for ROC coding is given by:

$$\text{Cov}(W_i, W_j) = \frac{\mu_W}{M-1}\left(\mu_W - \frac{2M+1}{3}\right) \tag{4.33}$$

4.5 Modulations vector function

Under the geometrical-ROC scheme that we consider in this paper, we want the modulations vector for $\mathbf{r}^1$ to be composed as a decreasing geometric sequence, starting from 1, and with rate m.

For (1.2), we denote $\Psi_R : \Omega \to \Xi_R$, the function for generating modulations vector for ROC.

It is parametized by the numbers $M \in \{1, \ldots, M\}$ and $m \in [0, 1]$.

We then define $\psi_{R,i}$ as:

$$\psi_{R,i}(\hat{\mathbf{w}}^k; m) = \psi_{R,i}((\hat{w}_1^k, \ldots, \hat{w}_M^k); m) = m^{M-\hat{w}_i^k} \tag{4.34}$$

such that

$$\Psi_R(\hat{\mathbf{w}}^k) = \Psi_R(\hat{w}_1^k, \ldots, \hat{w}_M^k) \tag{4.35}$$
$$= (\psi_{R,1}(\hat{w}_1^k, \ldots, \hat{w}_M^k), \ldots, \psi_{R,M}(\hat{w}_1^k, \ldots, \hat{w}_M^k)) \tag{4.36}$$
$$= (m^{M-\hat{w}_1^k}, \ldots, m^{M-\hat{w}_M^k}) = \mathbf{v}_R^l \tag{4.37}$$

with $l \in \{1, \ldots |\Omega_R|\}$.

Ψ_R is a bijective function so $|\Omega_R| = |\Lambda| = M!$.

For $k = 1$, we have that $\mathbf{r}^1 = (r_1^1 \; r_2^1 \; \ldots \; r_M^1)^T = (1 \; 2 \; \ldots \; M)^T$ then

$$\Psi_R(\hat{\mathbf{w}}^1) = (\psi_{R,1}(\hat{w}_1^1, \ldots, \hat{w}_M^1), \ldots, \psi_{R,M}(\hat{w}_1^1, \ldots, \hat{w}_M^1)) \tag{4.38}$$
$$= (m^{M-\hat{w}_1^1}, \ldots, m^{M-\hat{w}_M^1}) \tag{4.39}$$
$$= (m^{M-M}, \ldots, m^{M-1}) \tag{4.40}$$
$$= (m^0, m^1 \ldots, m^{M-1}) = \mathbf{v}_R^1 \tag{4.41}$$

Below, we consider the modulation vector being : $\mathbf{v}_{R,M}^1 = \Psi_R(\hat{\mathbf{w}}^1; m)$.

The modulation vector gated up to the first $I < M$ components is then defined by

$$\Psi_R(\hat{\mathbf{w}}^1) = (m^0, m^1 \ldots, m^{I-1}, 0, \ldots, 0) \tag{4.42}$$

4.6 $S_{R,M}$. Integration-Final Potential $I = M$

4.6.1 max($S_{R,M}$)

The maximum value of integration $S_{R,M}$ is defined in equation (1.31). Thus, for ROC coding, we have that $\mathbf{w}^1 = (M, M-1, \ldots, 1)$ and modulation vector is given by (4.41). Thus $\max(S_{R,M})$ is

$$=< \mathbf{w}^1, \mathbf{v}_{R,M}^1 >$$
$$= (M-0)m^0 + (M-1)m^1 + (M-2)m^2 + \ldots + (M-(M-1))m^{M-1}$$
$$= M(m^0 + m^1 + \ldots + m^{M-1}) - (m^1 + 2m^2 + \ldots + (M-1)m^{M-1}) \tag{4.43}$$
$$= M \sum_{i=0}^{M-1} m^i - \sum_{i=1}^{M-1} i\, m^i \tag{4.44}$$
$$= M\left(\frac{1-m^M}{1-m}\right) - \frac{1-m^{M+1}-(Mm^M+1)(1-m)}{(1-m)^2} \tag{4.45}$$

Simplifying, the max of integration for ROC is given by:

$$\max(S_{R,M}) = \frac{(1-m)(1+M)-(1-m^{M+1})}{(1-m)^2} \tag{4.46}$$

4.6.2 $\mathbf{E}[S_{R,M}]$, expectation of final potential

$$\begin{aligned}
\mathrm{E}[S_{R,M}] &= \mathrm{E}[\mathbf{v}_{R,M}^{\mathsf{T}}\ \mathbf{W}] = \mathbf{v}_{R,M}^{\mathsf{T}}\ \mathrm{E}[\mathbf{W}] && (4.47)\\
&= \mathbf{v}_{R,M}^{\mathsf{T}} \cdot \left(\frac{M+1}{2}, ..., \frac{M+1}{2}\right)^{\mathsf{T}} && (4.48)\\
&= (m^0\ m^1\ \ldots\ m^{M-1}) \cdot \left(\frac{M+1}{2}, ..., \frac{M+1}{2}\right)^{\mathsf{T}} && (4.49)\\
&= \left(\frac{m^0(M+1)}{2} + \frac{m^1(M+1)}{2} + ... + \frac{m^{M-1}(M+1)}{2}\right) &&\\
&= \frac{(M+1)}{2}(m^0 + m^1 + ... + m^{M-1}) && (4.50)\\
&= \frac{(M+1)}{2}\frac{1-m^M}{1-m} && (4.51)
\end{aligned}$$

Then the expectation of Integration $S_{R,M}$ is given by

$$\mathrm{E}[S_{R,M}] = \frac{(M+1)}{2}\frac{1-m^M}{1-m} \tag{4.52}$$

4.6.3 $\mathbf{Var}[S_{R,M}]$, variance of final potential

From (1.38), we have $\mathrm{Var}[S_{R,M}] = \mathbf{v}_{R,M}^{\mathsf{T}} \cdot \mathbf{K}_{WW}^{R} \cdot \mathbf{v}_{R,M}$. The Variance-covariance matrix, $\mathbf{K}_{WW}^{R}$ is given by (1.43), and the modulation vector $\mathbf{v}_{R,M}^{1}$ of final potential for geometric ROC coding is given by (4.41). Therefore:

$$
\begin{aligned}
&\mathrm{Var}[S_{R,M}] \\
&= \mathbf{v}_{R,M}^{\mathsf{T}} \cdot \mathbf{K}_{WW}^{R} \cdot \mathbf{v}_{R,M} \\
&= (m^0\ m^1\ \dots\ m^{M-1}) \begin{bmatrix} \mathrm{Var}W & \vartheta & \dots & \vartheta \\ \vartheta & \mathrm{Var}W & \dots & \vartheta \\ \vdots & \vdots & \ddots & \vdots \\ \vartheta & \vartheta & \dots & \mathrm{Var}W \end{bmatrix} \begin{bmatrix} m^0 \\ m^1 \\ \vdots \\ m^{M-1} \end{bmatrix} && (4.53)\\
&= m^0(m^0\mathrm{Var}W + m^1\vartheta + \dots + m^M\vartheta) + m^1(m^0\vartheta + m^1\mathrm{Var}W + \dots + m^M\vartheta) + && (4.54)\\
&\dots + m^{M-1}(m^0\vartheta + m^1\vartheta + \dots + m^{M-1}\mathrm{Var}W) \\
&= \mathrm{Var}W \sum_{i=0}^{M-1} m^{2i} + && (4.55)\\
&\vartheta\left[m^0\left(\sum_{i=0}^{M-1} m^i - m^0\right) + m^1\left(\sum_{i=0}^{M-1} m^i - m^1\right) + m^{M-1}\left(\sum_{i=0}^{M-1} m^i - m^{M-1}\right)\right] \\
&= \left(\frac{1-m^{2M}}{1-m^2}\right)\mathrm{Var}W + \left[\sum_{i=0}^{M-1} m^i\left(\frac{1-m^M}{1-m} - m^i\right)\right]\vartheta && (4.56)
\end{aligned}
$$

Using the geometric summation formulas, we get:

$$
\mathrm{Var}[S_{R,M}] = \left(\frac{1-m^{2M}}{1-m^2}\right)\mathrm{Var}W + \left[\left(\frac{1-m^M}{1-m}\right)^2 - \frac{1-m^{2M}}{1-m^2} - \right]\mathrm{Cov}(W_i, W_j) \tag{4.57}
$$

where $\mathrm{Var}W$ is given by (4.22) and $\mathrm{Cov}(W_i, W_j)$ by (4.33).

4.7 $S_{R,I}$. Integration-intermediate states, $I < M$

4.7.1 $\mathrm{E}[S_{R,I}]$, expectation at intermediate states

$$
\begin{aligned}
\mathrm{E}[S_{R,I}] &= \mathrm{E}[\mathbf{v}_{R,I}^{\mathsf{T}}\,\mathbf{W}] = \mathbf{v}_{R,I}^{\mathsf{T}}\,\mathrm{E}[\mathbf{W}] && (4.58)\\
&= \mathbf{v}_{R,I}^{\mathsf{T}} \cdot \left(\frac{(M+1)}{2}, ..., \frac{(M+1)}{2}\right)^{\mathsf{T}} \\
&= (m^0\ m^1\ \dots\ m^{I-1}\ 0\dots0) \cdot \left(\frac{(M+1)}{2}, ..., \frac{(M+1)}{2}\right)^{\mathsf{T}} \\
&= \left(\frac{m^0(M+1)}{2} + \frac{m^1(M+1)}{2} + ... + \frac{m^{I-1}(M+1)}{2}\right) \\
&= \frac{(M+1)}{2}(m^0 + m^1 + ... + m^{I-1}) \\
&= \frac{(M+1)}{2}\frac{1-m^I}{1-m} && (4.59)
\end{aligned}
$$

Then the expectation of Integration $S_{R,I}$ is given by

$$
\mathrm{E}[S_{R,I}] = \frac{(M+1)}{2}\frac{1-m^I}{1-m} \tag{4.60}
$$

4.7.2 $\mathbf{Var}[\boldsymbol{S_{R,I}}]$, variance at intermediate states

From (1.38), the variance at intermediate states is given by $\mathrm{Var}[S_{R,I}] = \mathbf{v}_{R,I}^{\mathsf{T}} \cdot \mathbf{K}_{WW}^{R} \cdot \mathbf{v}_{R,I}$. The Variance-covariance matrix $\mathbf{K}_{WW}^{R}$ is given by (1.43) and modulation vector $\mathbf{v}_{R,I}^{1}$ by (4.41). Therefore we have:

$$\begin{aligned}
&\mathrm{Var}[S_{R,I}] \\
&= \mathbf{v}_{R,I}^{\mathsf{T}} \cdot \mathbf{K}_{WW}^{R} \cdot \mathbf{v}_{R,I} && (4.61)\\
&= (m^0\ m^1\ \ldots\ m^{I-1}\ 0 \ldots 0) \begin{bmatrix} \mathrm{Var}W & \vartheta & \ldots & \vartheta \\ \vartheta & \mathrm{Var}W & \ldots & \vartheta \\ \vdots & \vdots & \ddots & \vdots \\ \vartheta & \vartheta & \ldots & \mathrm{Var}W \end{bmatrix} \begin{bmatrix} m^0 \\ m^1 \\ \vdots \\ m^{I-1} \\ 0 \\ \vdots \\ 0 \end{bmatrix} && (4.62)\\
&= m^0(m^0\mathrm{Var}W + m^1\vartheta + \ldots + m^I\vartheta) + m^1(m^0\vartheta + m^1\mathrm{Var}W + \ldots + m^I\vartheta) + && (4.63)\\
&\ldots + m^{I-1}(m^0\vartheta + m^1\vartheta + \ldots + m^{I-1}\mathrm{Var}W) \\
&= \mathrm{Var}W \sum_{i=0}^{I-1} m^{2i} + && (4.64)\\
&\vartheta \left[m^0 \left(\sum_{i=0}^{I-1} m^i - m^0 \right) + m^1 \left(\sum_{i=0}^{I-1} m^i - m^1 \right) + m^{I-1} \left(\sum_{i=0}^{I-1} m^i - m^{I-1} \right) \right] \\
&= \left(\frac{1 - m^{2I}}{1 - m^2} \right) \mathrm{Var}W + \left[\sum_{i=0}^{I-1} m^i \left(\frac{1 - m^I}{1 - m} - m^i \right) \right] \vartheta && (4.65)
\end{aligned}$$

Using the geometric summation formulas, we get:

$$\mathrm{Var}[S_{R,I}] = \left(\frac{1 - m^{2I}}{1 - m^2} \right) \mathrm{Var}W + \left[\left(\frac{1 - m^I}{1 - m} \right)^2 - \frac{1 - m^{2I}}{1 - m^2} - \right] \mathrm{Cov}(W_i, W_j) \qquad (4.66)$$

where $\mathrm{Var}W$ is given by (4.22) and $\mathrm{Cov}(W_i, W_j)$ by (4.33).

4.8 Behavior of discriminability for final potential

Let us assign $p = \dfrac{1 - m^{2M}}{1 - m^2}$, $q = \dfrac{1 - m^M}{1 - m}$ and substituting in the formulas,

$$\begin{aligned}
\max(S_{R,M}) &= \frac{(1 - m)(1 + M) - (1 - m^{M+1})}{(1 - m)^2} && (4.67)\\
&= \frac{1 + M}{1 - m} - \frac{(1 - m^{M+1})}{(1 - m)^2} && (4.68)\\
&= \frac{1 + M}{1 - m} - \frac{q + m^M}{1 - m} && (4.69)\\
&= \frac{M - q\, m}{1 - m} && (4.70)
\end{aligned}$$

$$\begin{aligned} \mathrm{E}[S_{F,M}] &= \lambda_F\ \mu_{W_F} && (4.71) \\ &= \frac{1-m^M}{1-m}\ \frac{M+1}{2} && (4.72) \\ &= q\ \frac{M+1}{2} && (4.73) \end{aligned}$$

$$\begin{aligned} \mathrm{Var}[S_{R,\mathcal{N}}] &= p\mathrm{Var}W\ + (q^2-p)\ \mathrm{Cov}(W_i, W_j) && (4.74) \\ &= p\ \mu_{W_R}\left(\frac{M-1}{6}\right) + (q^2-p)\frac{\mu_{W_R}}{M-1}\left(\mu_{W_R} - \frac{2M+1}{3}\right) && (4.75) \\ &= \frac{\mu}{6(M-1)}\left(p(M-1)^2 + 6(q^2-p)\left(\mu - \frac{2M+1}{3}\right)\right) && (4.76) \\ &= \frac{M+1}{12}(p\ M - q^2) && (4.77) \end{aligned}$$

then substituting in the formula of discriminability, max (4.70), expectation(4.73) and variance (4.77) of integration for ROC code, we get the following function,

$$\begin{aligned} D_F(M) &= \frac{\max(S_{F,M}) - \mathrm{E}[S_{F,M}]}{\sqrt{\mathrm{Var}[S_{F,M}]}} && (4.78) \\ &= \frac{\dfrac{M-q\ m}{1-m} - q\ \dfrac{M+1}{2}}{\sqrt{\dfrac{M+1}{12}(p\ M-q^2)}} && (4.79) \\ &= \sqrt{\frac{\dfrac{(2(M-q\ m) - q(M+1)(1-m))^2}{4(1-m)^2}}{\dfrac{M+1}{12}(p\ M-q^2)}} && (4.80) \\ &= \frac{\sqrt{3}}{1-m}\sqrt{\frac{(2(M-q\ m)-q(M+1)(1-m))^2}{(M+1)(p\ M-q^2)}} && (4.81) \end{aligned}$$

dividing numerator and denominator of the radicand by M^2 to find the tendency of the function when $M \to \infty$, we get,

$$D_F(M) = \frac{\sqrt{3}}{1-m}\sqrt{\frac{\left(2\left(1-\dfrac{q\ m}{M}\right) - q\left(1+\dfrac{1}{M}\right)(1-m)\right)^2}{\left(1+\dfrac{1}{M}\right)\left(p-\dfrac{q^2}{M}\right)}} \qquad (4.82)$$

Note that $\lim_{M\to\infty} p = \dfrac{1}{1-m^2}$ and $\lim_{M\to\infty} q = \dfrac{1}{1-m}$ then we have that,

$$\lim_{M\to\infty} D_F(M) = \lim_{M\to\infty} \frac{\sqrt{3}}{1-m}\sqrt{\frac{\left(2\left(1-\frac{q\,m}{M}\right)-q\left(1+\frac{1}{M}\right)(1-m)\right)^2}{\left(1+\frac{1}{M}\right)\left(p-\frac{q^2}{M}\right)}} \tag{4.83}$$

$$= \frac{\sqrt{3}}{1-m}\sqrt{\frac{(2-q(1-m))^2}{p}} \tag{4.84}$$

$$= \frac{\sqrt{3}}{1-m}\sqrt{1-m^2} \tag{4.85}$$

therefore for $m = 0.8$ the function $Y = D_F(M)$ has an asymptote horizontal in $Y = 5.2$ given that,

$$\lim_{M\to\infty} D_F(M) = \frac{\sqrt{3}}{1-0.8}\sqrt{1-0.8^2} = 5.196152422706633 \tag{4.86}$$

5 PEARSON CORRELATION COEFFICIENT

The scores of the Ranked-NoM, NoM and ROC codes are negatively correlated and the Pearson correlation coefficient ρ is the same for the three schemes. They are not independent since permutation of a given set of values implies correlation. That is, the correlation comes from the fact that the choice for, say the last value to be put at the last position of the vector, depends upon the value which has not been put into the vector yet.

The correlation coefficient is defined by

$$\rho = \frac{\text{Cov}(W_i, W_j)}{\sqrt{\text{Var}W_i}\sqrt{\text{Var}W_j}} \tag{5.1}$$

then we calculate ρ for each scheme. For the Ranked-NoM code,

$$\rho_H = \frac{\frac{\mu_W}{M-1}(\mu_W - \frac{2\mathcal{W}+1}{3})}{\left(\sqrt{\mu_W\frac{2\mathcal{W}+1}{3} - \mu_W^2}\right)^2} \tag{5.2}$$

$$= \frac{\frac{1}{M-1}\left(\mu_W - \frac{2\mathcal{W}+1}{3}\right)}{\frac{2\mathcal{W}+1}{3} - \mu_W} \tag{5.3}$$

$$= \frac{-1}{M-1} \tag{5.4}$$

for the ROC code,

$$\rho_R = \frac{\dfrac{\mu_W}{M-1}(\mu_W - \dfrac{2M+1}{3})}{\left(\sqrt{\dfrac{\mu_X}{6}(M-1)}\right)^2} \tag{5.5}$$

$$= \frac{\dfrac{1}{M-1}\left(\mu_W - \dfrac{2M+1}{3}\right)}{\dfrac{1}{6}(M-1)} \tag{5.6}$$

$$= \frac{2(3\mu_W - 2M - 1)}{(M-1)^2} \tag{5.7}$$

$$= \frac{-1}{M-1} \tag{5.8}$$

and for NoM, ρ is defined by,

$$\rho_F = \frac{\mu_W\left(\dfrac{\mathcal{W}-1}{M-1}\right) - \mu_W^2}{(\sqrt{\mu_W(1-\mu_W)})^2} \tag{5.9}$$

$$= \frac{\left(\dfrac{\mathcal{W}-1}{M-1}\right) - \mu_W}{1-\mu_W} \tag{5.10}$$

$$= \frac{-1 + \dfrac{\mathcal{W}}{M}}{(M-1)\left(1 - \dfrac{\mathcal{W}}{M}\right)} \tag{5.11}$$

$$= \frac{-1}{M-1} \tag{5.12}$$

therefore, $\rho_H = \rho_R = \rho_F = \dfrac{-1}{M-1}$ thus the correlation vanishes when $M- > \infty$, and it is because the effect of previous choice tends to zero for next choice. So, it is the fact that we make permutations of a given set of values.
